# Integration of meta-analysis and network pharmacology analysis to investigate the pharmacological mechanisms of traditional Chinese medicine in the treatment of hepatocellular carcinoma

**DOI:** 10.3389/fphar.2024.1374988

**Published:** 2024-03-15

**Authors:** Jie Lin, Huaijuan Guo, Hanjiao Qin, Xuewen Zhang, Jiyao Sheng

**Affiliations:** ^1^ Department of Hepatobiliary and Pancreatic Surgery, The Second Hospital of Jilin University, Changchun, Jilin, China; ^2^ Department of Oncology, The Affiliated Hospital of Yangzhou University, Yangzhou, Jiangsu, China

**Keywords:** hepatocellular carcinoma, traditional Chinese medicine, meta-analysis, network pharmacology analysis, therapeutic value

## Abstract

**Background:** This study will explore the therapeutic value of traditional Chinese medicine (TCM) in Hepatocellular Carcinoma (HCC) through meta-analysis, combined with network pharmacology analysis.

**Methods:** The results of randomized controlled trials on TCM and HCC were retrieved and summarized from multiple databases. The effective active com-pounds and target genes of the high-frequency TCM were obtained using the TCMSP database, and disease targets of HCC were acquired through the public disease database. The network pharmacology analysis was used to get the core genes and investigate the potential oncogenic molecular mechanism.

**Results:** A total of 14 meta-analysis studies with 1,831 patients suggested that therapy combined TCM is associated with better clinical efficacy and survival prognosis, as well as avoiding many adverse events. A total of 156 compounds, 247 herbal target genes and 36 core genes were identified. The function analysis suggested above genes may participate development in HCC through regulating some pathways, such as HIF-1 pathway and PD-L1 immune-related pathway.

**Conclusion:** TCM, as a novel, safe, and effective multi-mechanism therapy, holds greater value in the treatment of HCC.

## 1 Introduction

The incidence of liver cancer is steadily increasing, with an estimated one million cases expected by 2025. Among liver cancer types, 90% are HCC (Llovet et al., 2021). HCC ranks as the second leading cause of cancer-related deaths worldwide (Llovet et al., 2022), and approximately six million individuals have lost their lives due to HCC (Shen et al., 2023).

Currently, the primary treatment for HCC involves surgery, often supplemented with chemotherapy, radiotherapy, targeted therapies, and immune-based treatments. Despite the array of available treatment options for HCC, the overall prognosis for HCC patients has not shown significant improvement, and treatment outcomes can vary considerably from person to person (Njei et al., 2015). Factors such as the high recurrence rate of HCC after surgical interventions (Liu and Song, 2021) and the development of resistance to chemotherapy drugs (Tang et al., 2020) contribute to the challenging prognosis of HCC.

HCC treatment has now entered the era of comprehensive therapy, and Traditional Chinese Medicine (TCM) has demonstrated promising results in several clinical studies. Chinese HCC diagnosis and treatment guidelines recognize Huai Er granule as an important method for postoperative adjuvant therapy for HCC, with studies confirming its effectiveness (Chen et al., 2018). TCM, with its diverse components, can simultaneously target multiple aspects of tumor biology, exerting anti-tumor effects. Traditional experimental approaches often struggle to elucidate the specific mechanisms of TCM’s action. In recent years, the accumulation of biological big data and the advancement of bioinformatics have provided hope for a better understanding of the intricate mechanisms underlying TCM’s anti-tumor effects. This study will leverage the strengths of meta-analysis and network pharmacology analysis to explore the potential pharmacological mechanisms of TCM in treating HCC.

## 2 Material and methods

### 2.1 Literature search stragety

The Preferred Reporting Items for Systematic Reviews and Meta-Analyses (PRISMA) guidelines were employed to guide the conduct and reporting of this meta-analysis. JL and HG conducted systematic searches of six databases (PubMed, Embase, Cochrane Library, Chinese Biomedical Literature (CBM) database, China National Knowledge Infrastructure (CNKI) database, and Wanfang database) until 1 October 2023. The complete search strategy was in the Supplementary Material S1.

### 2.2 Criteria of eligibility

Details of the inclusion and exclusion criteria are shown in Table 1.

**TABLE 1:** Article inclusion and exclusion criteria.

PICOS	Inclusion criteria	Exclusion criteria
Participants	1 Age 18 or older	1 Younger than 18 years
	2 Conformed to the diagnostic criteria of “Chinese Society of Clinical Oncology Guidelines for the Diagnosis and Treatment of Primary Liver Cancer	2 Complicated by serious primary cardiovascular, renal, hemopoietic, immune or mental diseases
	3 Patients with Barcelona Clinic Liver Cancer (BCLC) staging B or C, Child–Pugh A or B and be fitting to take intervention therapy	3 Those who failed to follow up or with incomplete data during observation
	4 Estimated survivals ≥ 3 months	4 Estimated survivals < 3 months
Intervention	The intervention group was treated with TCM therapy including oral TCM decoction and Chinese patent medicine combined with TACE or chemotherapy	The intervention group was treated with acupuncture, tuina, or acupoint application and other external therapies of Chinese medicine
Comparison	The control group was treated with conventional therapy	The control group was treated with TCM treatment
Outcome	Overall response rate (ORR); Disease control rate (DCR); Overall survival (OS); Progression-free survival (PFS); Recurrence-free survival (RFS); Disease- free survival (DFS); adverse events (AEs)	Incomplete or unidentified data
Study design	Randomized controlled trial (RCT)	Non-RCTs
Others	None	Duplicate publications, abstracts, reviews, case reports, and letters

ORR, was defined as the proportion of patients with complete response (CR)+ partial response (PR) after treatment to the total number of patients. DCR, was defined as the percentage of patients who achieved response (PR + CR) + stable disease (SD) after treatment to the total number of patients.

### 2.3 Data extraction and quality evaluation

In this study, two researchers (JL and HG) independently performed a literature search and screening process adhering to the aforementioned inclusion and exclusion criteria. They meticulously extracted crucial information from the selected studies, including the first author’s name, publication year, geographic region of the study, sample size, cancer type under investigation, the employed detection method (qRT-PCR or RNA-seq), outcome measures assessed, the duration of follow-up in months, as well as HR and their corresponding 95% CI pertaining to the prognostic indicators analyzed. JL and HG, independently performed data extraction and conducted a rigorous assessment of the included studies’ quality. If there are any discrepancies, they sought resolution through consultation with a third evaluator. We extracted basic information from the included literature, including first author, sample size, age or sex, clinical status, management measures, TCM protocols, and treatment outcomes. The bias of included studies was independently assessed according to the Cochrane risk bias tool in the Cochrane Handbook for Systematic Reviews of Interventions (Cumpston et al., 2019).

### 2.4 Statistical analysis

RevMan 5.2 software and STATA 12 were used for data analysis and processing of meta. For dichotomous variables, odds ratio (OR) was used as the effect size index. For continuous variables, the mean difference (MD) was used as an effect size indicator and 95% CI was indicated in the forest map. If there is heterogeneity between the two groups (*p* < 0.1 or I^2^ > 50%), a random effects model is used. Otherwise, the fixed effect model is adopted. Subgroup analysis was performed according to the type of OS and AEs. Sensitivity analysis was performed using Stata SE12.0 software. Publication bias was visually assessed using funnel plots in RevMan 5.2 software.

### 2.5 Network pharmacology study of effective TCM components in HCC

The TCM prescriptions in the results of meta-analysis were ranked based on their total occurrences, and those appearing four or more times were taken as the main research targets, which were Dangshen, Fuling, Chaihu, Baizhu, Banzhilian, Danggui, Gancao, Baishao and Huangqi. We obtained the bioactive components and corre-sponding drug targets of TCM from Traditional Chinese Medicine Systems Pharma-cology Database and Analysis Platform (TCMSP, https://tcmspw.com/tcmsp.php) (Ru et al., 2014). Oral bioavailability (OB) refered to the drug concentration and rate at which a TCM ingredient circulates through the body, and drug similarity (DL) refered to the correlation with known compounds, we used OB ≥ 30% and DL ≥ 0.18 as screening criteria to screen nine TCM active components (Liu et al., 2013). Then from The universal Protein Database (Uniprot, https://www.uniprot.org/) to download The human genetic information about these ingredients targets for gene annotations (Bairoch et al., 2005).

### 2.6 Identify the disease targets of HCC

Our team utilized “hepatocellular carcinoma” as the search keyword and retrieved disease targets related to HCC from databases including DisGeNet (https://www.disgenet.org/), GeneCards (https://www.genecards.org/), OMIM (http://omim.org/), TTD (http://db.idrblab.net/ttd/), and CTD (https://ctdbase.org/).

### 2.7 Acquisition of TCM -HCC intersection gene and construction of protein and protein interaction network

The vene package was used to identify the intersecting genes of TCM and HCC, and then the intersecting genes were imported into the STRING database (https://string-db.org/) (Szklarczyk et al., 2019), Human was selected as the genus, score >0.4 and hidden independent protein molecules were used as the screening conditions. The above results were imported into Cytoscape3.8.2 (Doncheva et al., 2019).

### 2.8 Identification of TCM -HCC core genes

CytoNCA plugin calculated 6 parameters: betweenness (BC), closeness (CC), degree (DC), eigenvector (EC), local average connectivity-base method (LAC) and Network (Tang et al., 2015). The genes above the mean value were extracted by two calculations and used as core genes.

### 2.9 Enrichment analysis of GO and KEGG pathways

In order to better observe the enrichment pathways of drugs and HCC genes, we performed functional enrichment analysis of important gene clusters. GO analysis (gene ontology), including (biological process, BP), (cell component, CC) and (molecular function, MF) three parts, KEGG signaling pathway analysis and DO (disease ontology) analysis.

## 3 Results

### 3.1 Identification and selection

**FIGURE 1:**
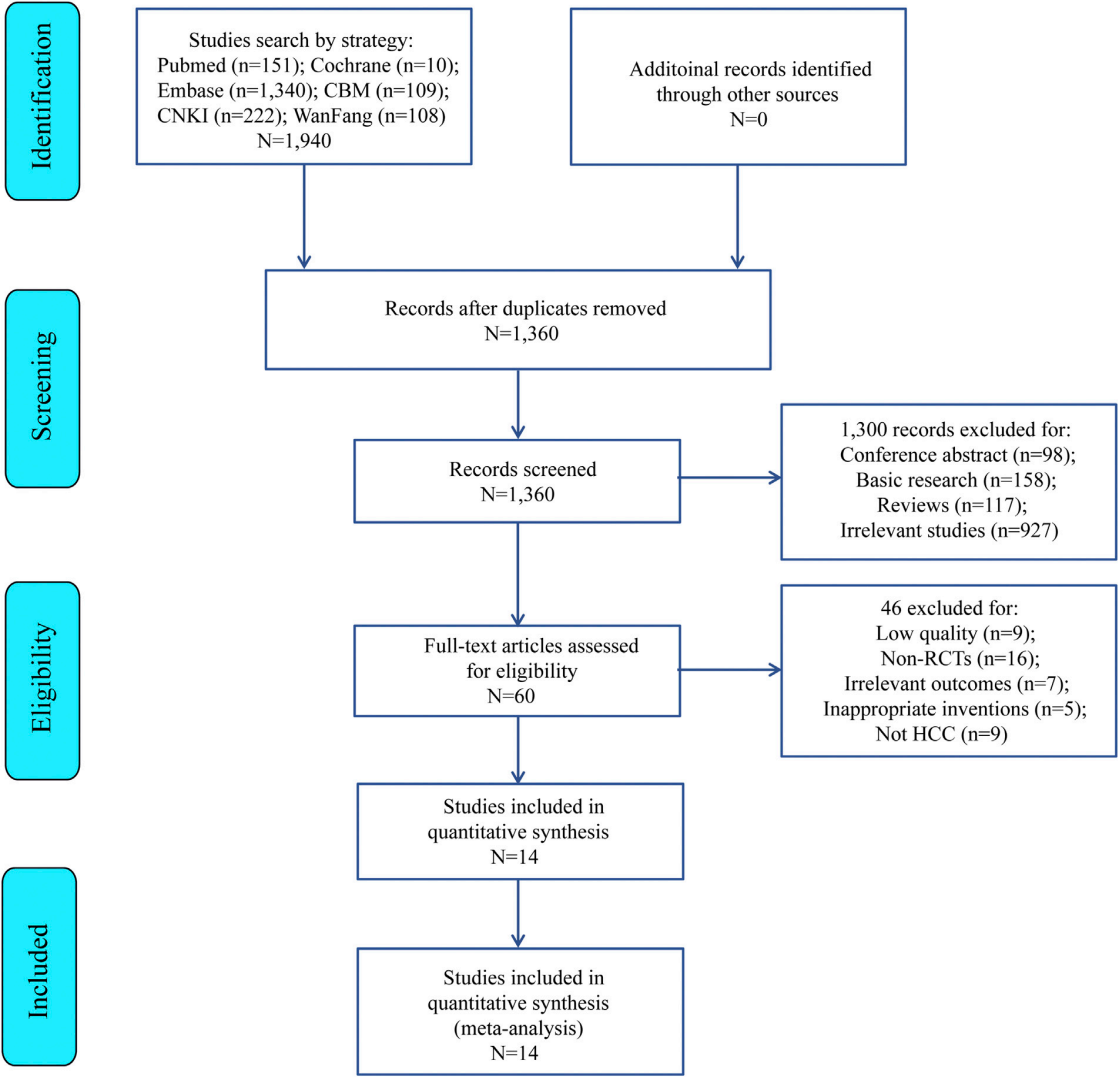
Flow chart of this study.

Six databases (PubMed = 151, Cochrane = 10, Embase = 1,340, CBM = 109, CNKI = 222, and WanFang = 108) were searched to obtain 1,940 candidate literatures, of which 1,360 only remained after removing duplicates. Then, additional 1,300 literatures were excluded due to irrelevant titles or abstracts. Finally, the remaining 60 articles were excluded by our eligibility criteria, and finally 14 randomized controlled trials (RCTS) (Han et al., 1997; Xie et al., 2008; Hou and Lu, 2009; Wang et al., 2009; Tian et al., 2010; Chen et al., 2012; Yang et al., 2013; Gao, 2014; Xu et al., 2014; JING, 2015; Zhai et al., 2018; Zhang et al., 2019; Yang et al., 2021; Tong et al., 2022) (Figure 1) met the meta inclusion criteria. Table 2 lists the main features of these 14 included articles.

**TABLE 2:** Characteristics of the 14 studies included in the meta-study.

First author	Year	Sample size (T/C,n)	Mean age or age range (T/C)	Gender [T (M/F), C (M/F)]	Clinical status	Common treatment (regimen)	TCM intervention	Control intervention	Main outcome
Chen et al. (2012)	2012	120 (60/60)	48.54 ± 8.83/48.67 ± 8.76	60(56/4), 60(58/2)	Not mentioned	TACE	Jiedu Granules Combined with Cinobufacini Injection	No additional Tx	PFS; OS
Gao (2014)	2014	58 (30/28)	43.53 ± 12.51/45.23 ± 10.51	30(17/13), 28(13/15)	KPS≥60	FOLFOX4	Ganfule prescription	No additional Tx	ORR; DCR; OS
Han et al. (1997)	1997	60 (30/30)	39-59/37-64	30(24/6), 30(26/4)	Not mentioned	Move stripe field radiation	Xuefu zhuyu decoction	Placebo	OS
Hou and Lu (2009)	2009	67 (35/32)	33-69/34-72	35(27/8), 32(25/7)	KPS≥70	TACE	TCM	No additional Tx	ORR; DCR; AEs
Jing (2015)	2015	106 (53/53)	56.60 ± 10.39/58.70 ± 11.86	Not mentioned	KPS≥60	TACE	TCM	No additional Tx	ORR; DCR; AEs
Tian et al. (2010)	2010	97 (49/48)	51.44 ± 10.5/52.37 ± 10.81	49(40/9), 48(41/7)	KPS≥60	chemotherapy	TCM	No additional Tx	ORR; DCR; AEs
Wang et al. (2009)	2009	77 (40/37)	51.67 ± 10.28/52.40 ± 10.61	40(33/7), 37(32/5)	KPS≥60	TACE	Ganji Recipe and Fructus Bruceae Oil Emulsion	No additional Tx	ORR; DCR; OS; AEs
Wu et al. (2022)	2022	72 (36/36)	44.5 ± 3.2/43.9 ± 3.3	36(26/10), 36(30/6)	KPS≥70	TACE	Fuzheng Jiedu Xiaoji formula	No additional Tx	ORR; DCR
Xie et al. (2008)	2008	122 (61/61)	Not mentioned	Not mentioned	KPS≥60	chemotherapy	Jinlong capsule	No additional Tx	OS
Xu et al. (2014)	2014	108 (54/54)	58 ± 10/57 ± 11	54(39/15), 54(40/14)	Not mentioned	TACE	Modified Chaishao Liujunzi decoction	No additional Tx	ORR; DCR; AEs; OS
Yang et al. (2013)	2013	70 (34/36)	Not mentioned	Not mentioned	KPS≥60	E-ADM+5-FU + DDP TACE	Jin-long capsules	No additional Tx	ORR; DCR
Yang et al. (2021)	2021	291 (144/147)	53.81 ± 7.61/55.08 ± 9.68	144(120/24), 147(123/24)	Not mentioned	TACE	Fuzheng Jiedu Xiaoji formulation	No additional Tx	OS; PFS
Zhai et al. (2018)	2018	364 (180/184)	Not mentioned	180(147/33), 184(164/20)	ECOG≤3	TACE	TCM	No additional Tx	RFS; OS
Zhang et al. (2019)	2019	219 (107/112)	56.74 ± 8.43/55.24 ± 10.83	107(78/29), 112(71/41)	Not mentioned	TACE	Ganji Formulation	Placebo	OS; DFS; AEs

### 3.2 Assessment of risk of bias

**FIGURE 2:**
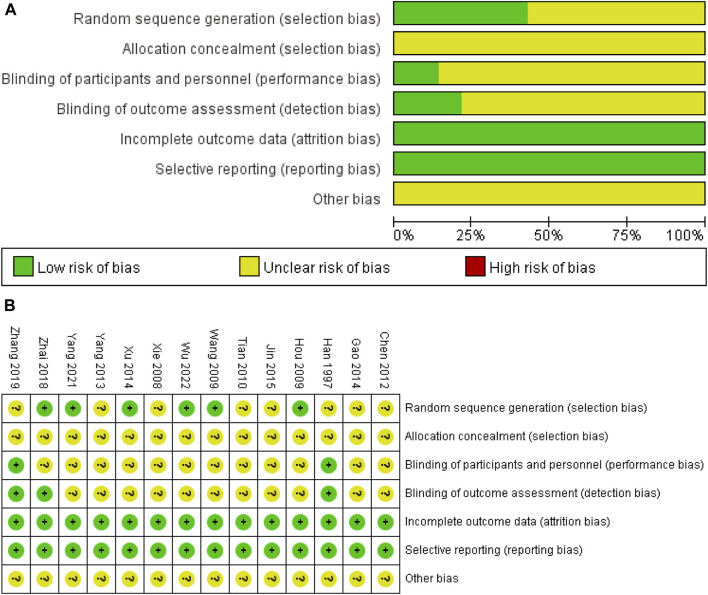
**(A)** classification of bias risk of included articles and **(B)** bias characteristics of each included article.

The results of the risk of bias assessment were shown in Figure 2. All the included studies were randomized, and six studies were considered to have a low risk of bias by using a random number table method (Hou and Lu, 2009; Wang et al., 2009; Xu et al., 2014; Zhai et al., 2018; Yang et al., 2021; Tong et al., 2022). Studies that do not mention specific randomized methods were defined as having an unclear risk of bias. Also, two studies (Han et al., 1997; Zhang et al., 2019) mentioned double-blinding, one study (Zhai et al., 2018) mentioned blinding of outcome assessors, the remaining studies did not report blinding of investigators, patients, and outcome evaluators. All studies completed data collection and all reports, so the risk was considered low. Although there were other biases in the study that made some of the risk of bias unclear, these potential biases were also not reported in the paper.

### 3.3 Overall response rate and disease control rate

**FIGURE 3:**
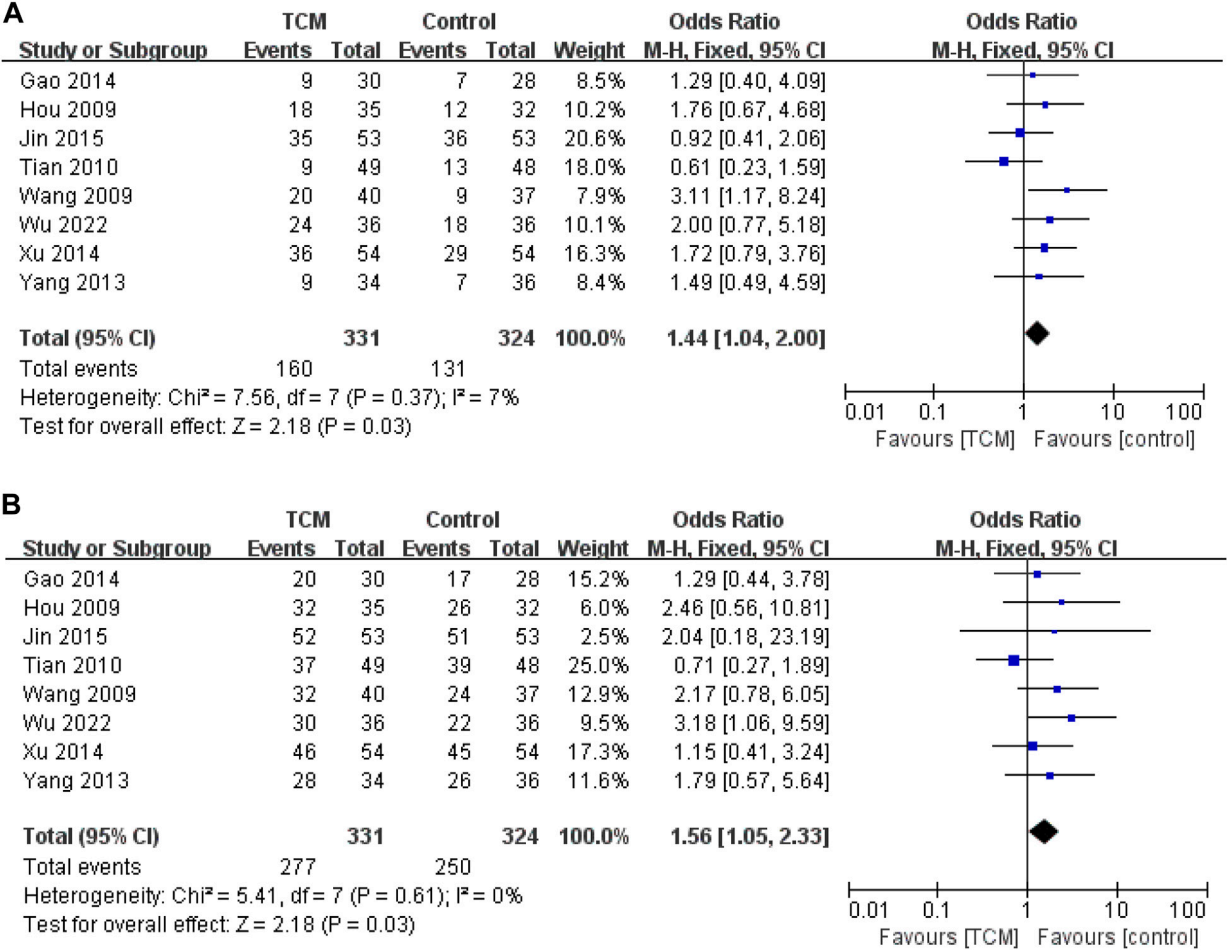
**(A)** ORR between TCM group and control group, **(B)** DCR between TCM group and control group.

Eight of the included studies mentioned Overall response rate (ORR) and Disease control rate (DCR) data. (Hou and Lu, 2009; Wang et al., 2009; Tian et al., 2010; Yang et al., 2013; Gao, 2014; Xu et al., 2014; JING, 2015; Tong et al., 2022). In HCC patients, the ORR in the combined treatment group was better than that in the non-combined group (OR = 1.44; 95% confidence intervals (CI) 1.04-2.00, *p* = 0.03) (Figure 3A). The DCR of the combined treatment group was also higher than that of the control group (OR = 1.56; 95% CI 1.05-2.33, *p* = 0.03) (Figure 3B).

### 3.4 Multi-survival analysis

**FIGURE 4:**
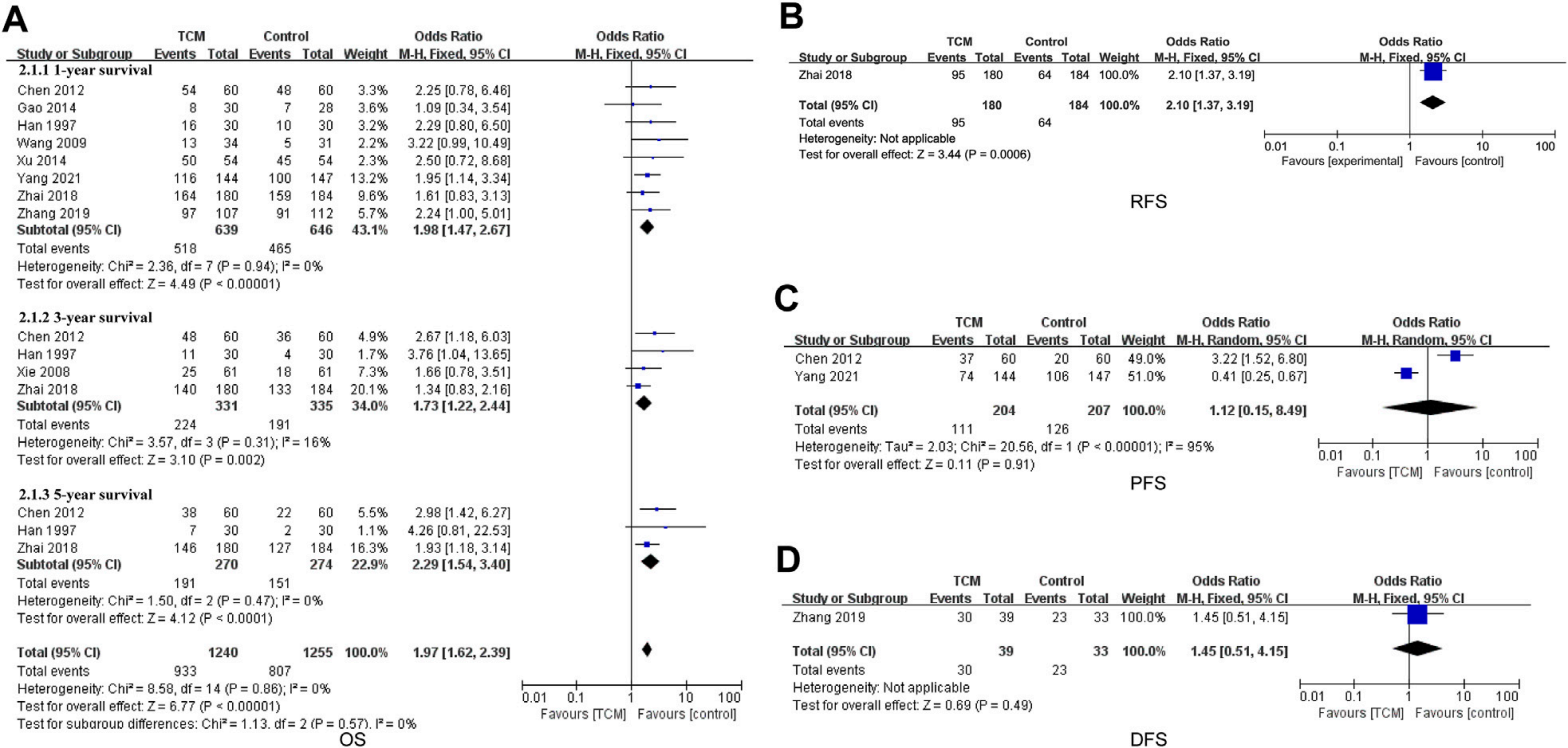
**(A)** Forest plot and subgroup analysis of OS comparison between TCM and control group, **(B)** forest plot of PFS comparison between TCM and control group, **(C)** forest plot of RFS comparison between TCM and control group, and **(D)** forest plot of DFS comparison between TCM and control group.

Nine included studies mentioned overall survival (OS) data (Han et al., 1997; Xie et al., 2008; Wang et al., 2009; Chen et al., 2012; Gao, 2014; Xu et al., 2014; Zhai et al., 2018; Zhang et al., 2019; Yang et al., 2021) (Figure 4A). The pooled results showed that combined treatment with TCM improved the OS of HCC patients (OR = 1.97, 95% CI 1.62−2.39, *p* < 0.00001). Subgroup analysis suggested 1-year survival (OR = 1.98, 95%CI 1.47-2.67, *p* < 0.00001), 3-year survival (OR = 1.73, 95%CI 1.22-2.44, *p* = 0.002) and 5-year survival (OR = 2.29, 95% CI 1.54−3.40, *p* < 0.0001) were significant.

One study (Zhai et al., 2018) reported results for Recurrence-free survival (RFS). The results showed that the RFS were significantly longer in the TCM group than in the control group (OR = 2.10, 95% CI 1.37-3.19, *p* = 0.0006) (Figure 4B).

Two study (Chen et al., 2012; Yang et al., 2021) involved data on progression-free survival (PFS). This result indicated that there was no change between the TCM group and the control group (OR = 1.12, 95%CI 0.15-8.49, *p* = 0.91) (Figure 4C). One study (Zhang et al., 2019) referred to disease-free survival (DFS) data, which showed no difference between the TCM group and the control group (OR = 1.45, 95% CI 0.51-4.15, *p* = 0.49) (Figure 4D).

### 3.5 Adverse events

**FIGURE 5:**
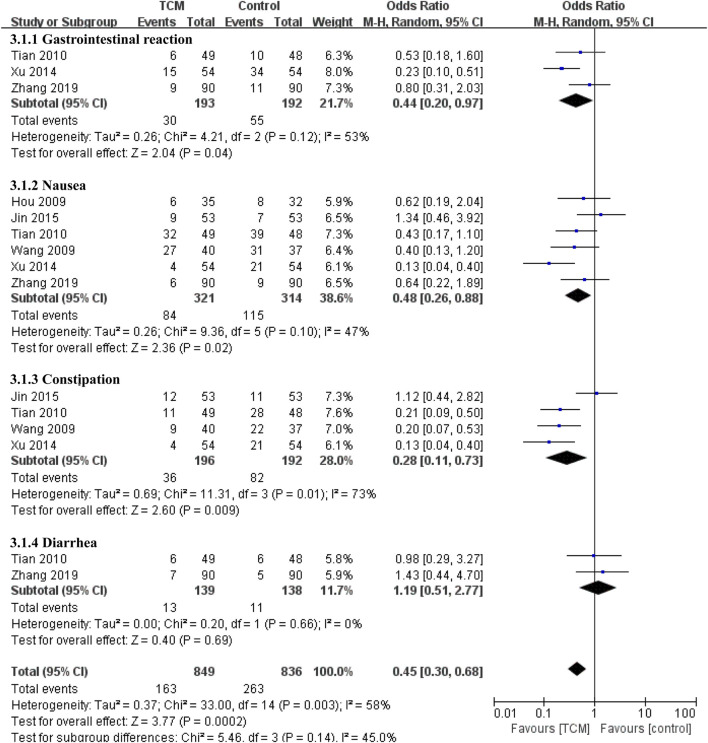
Forest plot and subgroup analysis of gastrointestinal adverse events.

Digestive complications were reported in six studies (Hou and Lu, 2009; Wang et al., 2009; Tian et al., 2010; Xu et al., 2014; JING, 2015; Zhang et al., 2019) (Figure 5), Compared with the control group, the combined TCM group reduced the incidence of gastrointestinal reactions (OR = 0.44, 95% CI 0.20-0.97, *p* = 0.04), nausea (OR = 0.48, 95% CI 0.26-0.88, *p* = 0.02) and constipation (OR = 0.28, 95% CI 0.11-0.73, *p* = 0.009). As for diarrhea, the results showed no significant difference between the two groups (OR = 1.19, 95% CI 0.51-2.77, *p* = 0.69), which may be due to the small number of data included.

**FIGURE 6:**
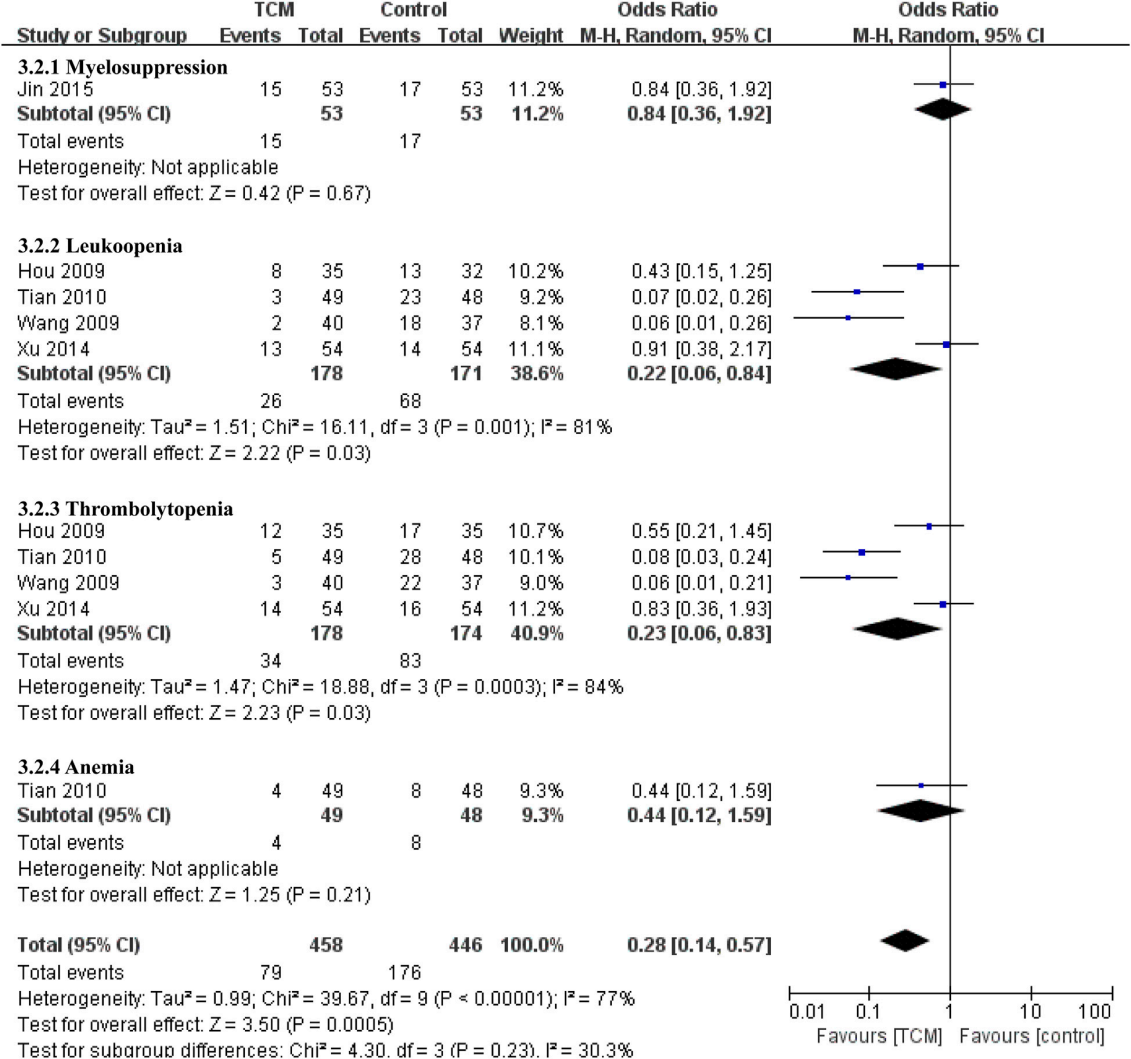
Forest plot and subgroup analysis of hematological adverse reactions.

Five studies involved complications of the blood system (Hou and Lu, 2009; Wang et al., 2009; Tian et al., 2010; Xu et al., 2014; JING, 2015) (Figure 6), The results showed that TCM group was superior to control group in preventing leukopenia (OR = 0.22, 95% CI 0.06-0.84, *p* = 0.03) and thrombocytopenia (OR = 0.23, 95% CI 0.06-0.83, *p* = 0.03). There was no effect on myelosuppression (OR = 0.84, 95% CI 0.36-1.92, *p* = 0.67) and anemia (OR = 0.44, 95% CI 0.12-1.59, *p* = 0.21).

**FIGURE 7:**
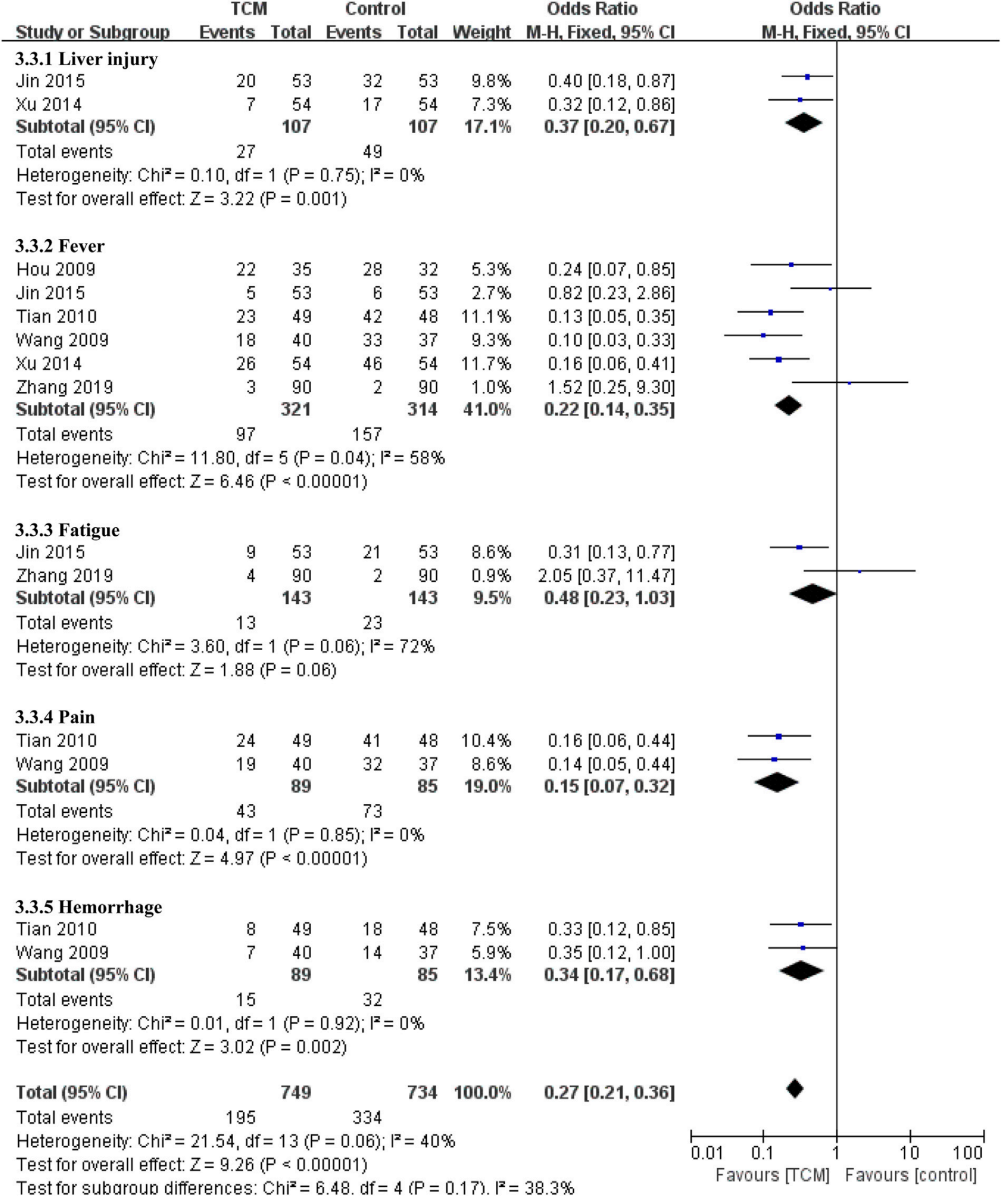
Forest plots and subgroup analyses for other adverse events.

As for other adverse events. Compared with the control group, the TCM group had benefit in reducing liver injury (OR = 0.37, 95% CI 0.20-0.67, *p* = 0.001). Alao, there are obvious effect on decreasing the incidence of fever (OR = 0.22, 95% CI 0.14-0.35, *p* < 0.00001), pain (OR = 0.15, 95% CI 0.07−0.32, *p* < 0.00001) and hemorrhage (OR = 0.34, 95% CI 0.17−0.68, *p* = 0.002), but There was no significant difference between two groups in the incidence of fatigue (OR = 0.48, 95% CI 0.23-1.03, *p* = 0.06) (Figure 7).

### 3.6 Sensitivity analysis and publication bias

**FIGURE 8:**
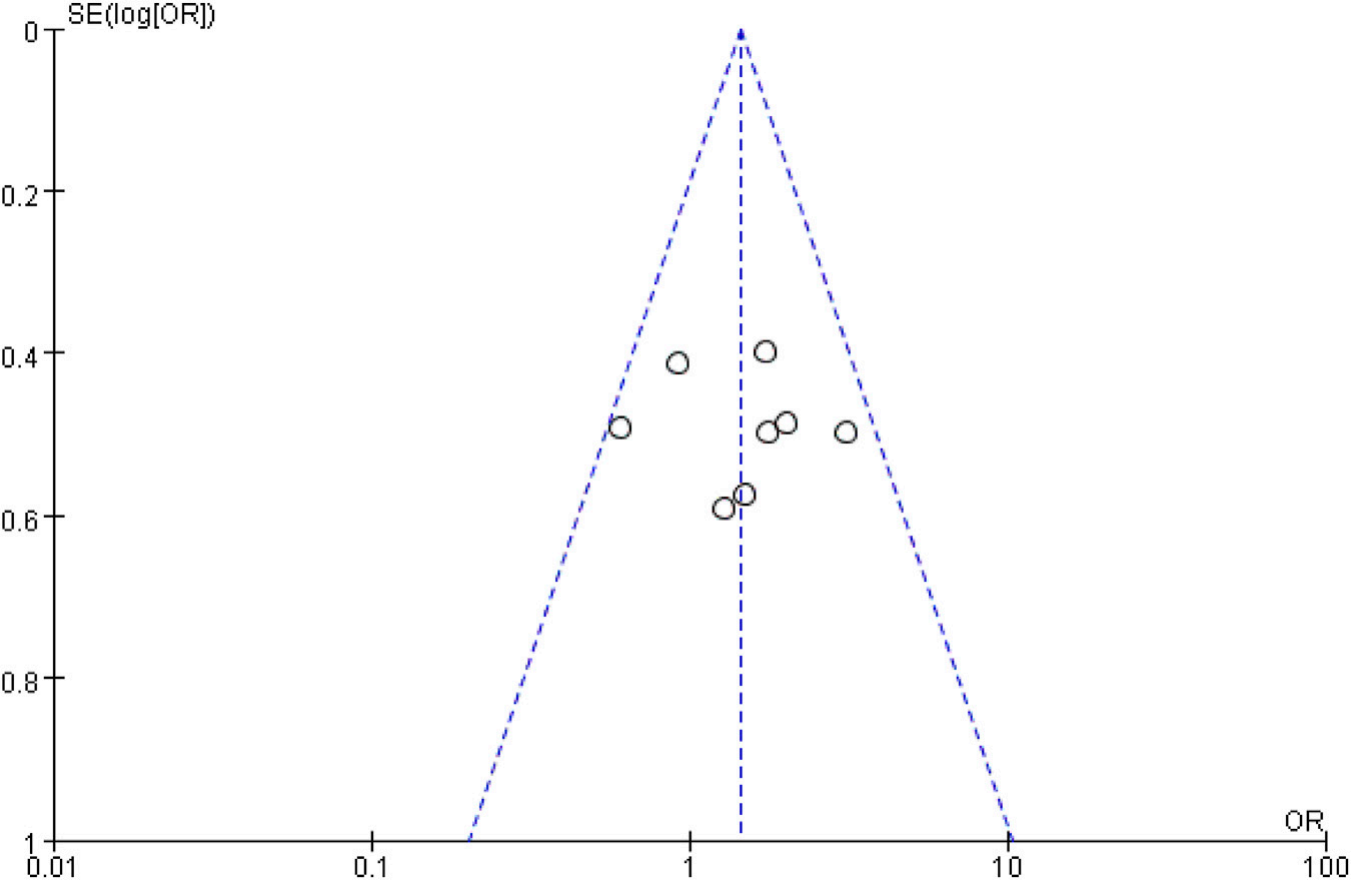
Funnel diagram of ORR.

In the sensitivity analysis (Supplementary Figure S1), the pooled ORs of other factors were not significantly affected by any single study, indicating the stability of the results. For ORR, a funnel plot was used to assess publication bias (Figure 8), and the results suggested basic symmetry on the two sides of the funnel, indicating no significant publication bias.

### 3.7 Effective herbs extraction

TCM components included in the study were ranked by counts ≥4, the most effective herbs included: Dangshen, Fuling, Chaihu, Baizhu, Banzhilian, Danggui, Gancao, Baishao, Huangqi (Table 3).

**TABLE 3:** Characteristics of high-frequency Chinese herbs.

Pharmaceutical name	Chinese name	Counts	Frequency 1 (counts/total herb counts) (%)	Frequency 2 (counts/study numbers) (%)
Codonopsis pilosula	Dangshen	8	5.44	57.14
Poria cocos	Fuling	7	4.76	50.00
Radix Bupleuri	Chaihu	7	4.76	50.00
Macrocephala	Baizhu	6	4.08	42.86
Scutellaria barbata	Banzhilian	5	3.40	35.71
Angelica sinensis	Danggui	5	3.40	35.71
Liquorice	Gancao	5	3.40	35.71
Radix paeoniae alba	Baishao	5	3.40	35.71
Astragalus	Huangqi	4	2.72	28.57

### 3.8 Constructs of TCM-HCC genes and drug-component-target

**FIGURE 9:**
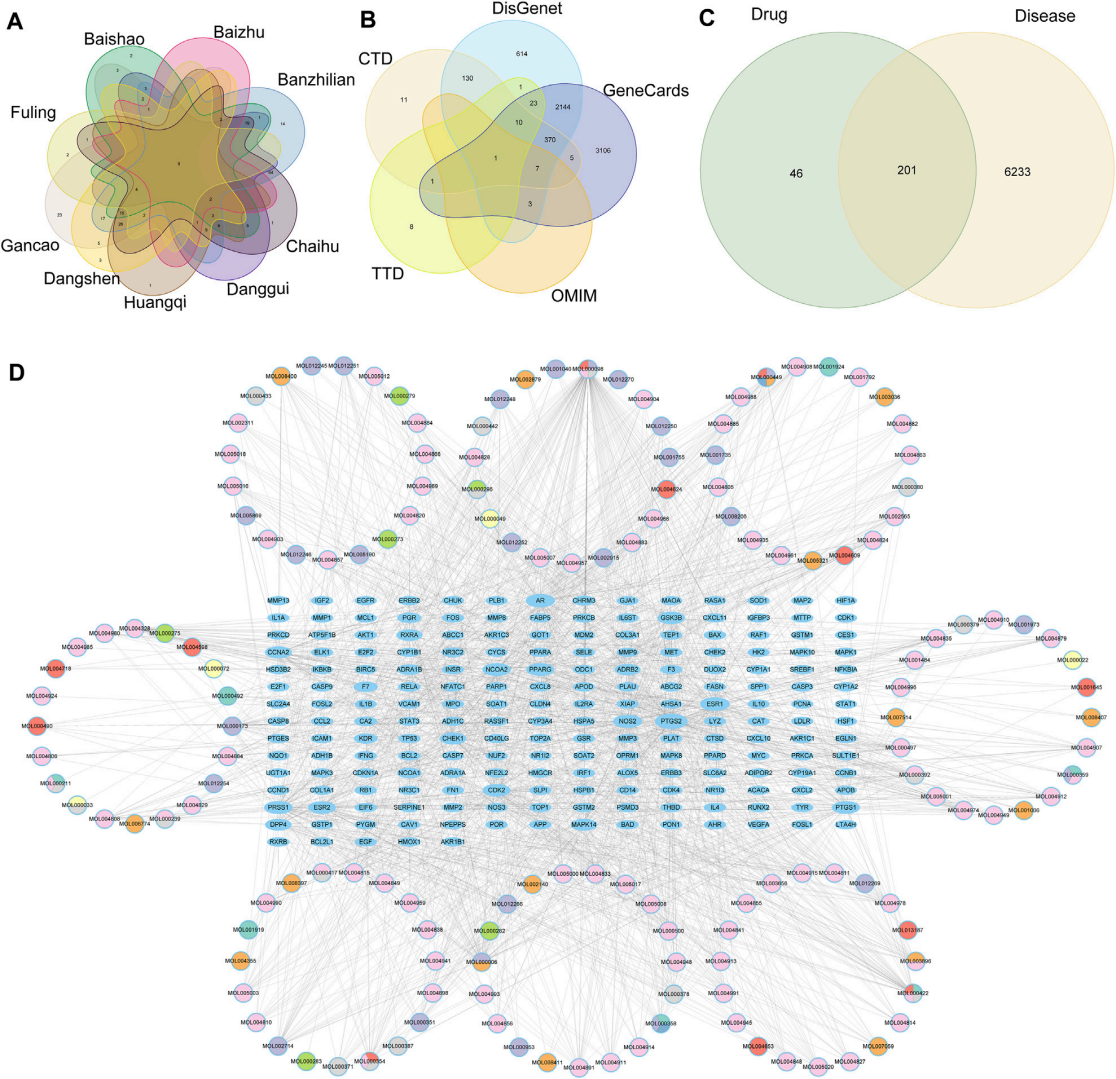
**(A)** vene diagram of gene targets of high-frequency Chinese herbs, **(B)** Multiple databases of disease targets for HCC, **(C)** vene graph of TCM targets and HCC targets, **(D)** Network of herb components an TCM-HCC targets.

Through searching the prescription database, we found that nine effective Chinese medicines contained 156 compounds and 247 target genes of Chinese medicines. We drew the map of these drug target genes (Figure 9A), and we found that there was intersection between the targets, which meant that multiple TCM may act on the same genes.

A total of 6,434 disease-related targets associated with HCC were retrieved from five public disease databases (Figure 9B). Through Venn diagram intersection analysis, 201 drug-disease targets were identified and selected (Figure 9C). Subsequently, these TCM-HCC targets were imported into the STRING database, resulting in a network with 201 nodes, 4,299 edges, and a PPI enrichment *p*-value ≤1.0e-16 (Supplementary Figure S2). Finally, a network graph of TCM components and TCM-HCC targets was constructed using Cytoscape (Figure 9D), details of the drug network were in Supplementary Table S1.

### 3.9 Identification and functional enrichment analysis of core genes

**FIGURE 10:**
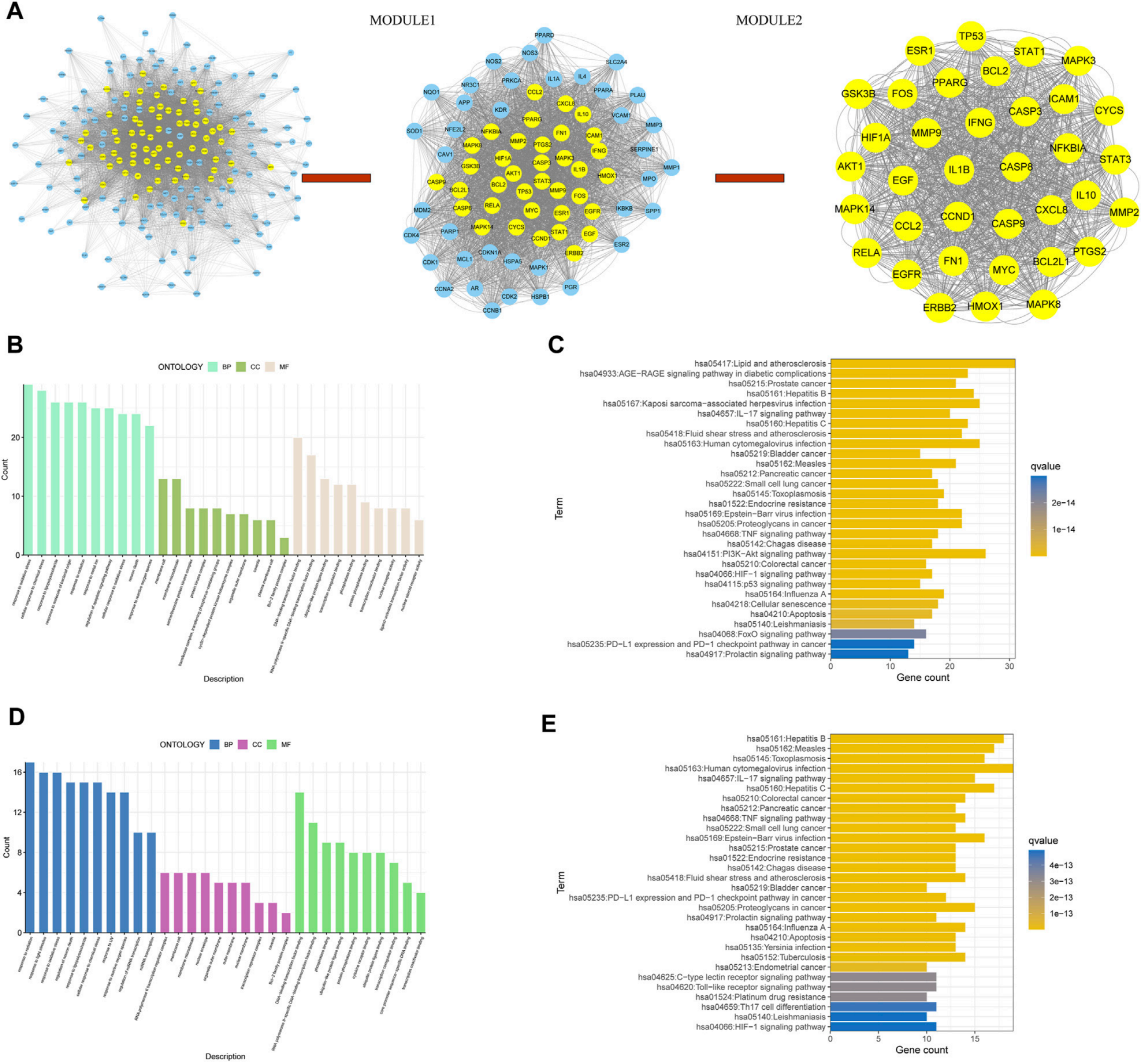
**(A)** On the left, PPI networks of cross genes between TCM targets and disease targets; In the middle, PPI network of module1 gene; On the right, PPI network of module2 gene, **(B)** GO analysis of module1 gene, **(C)** KEGG analysis of module1 gene, **(D)** GO analysis of module2 gene, and **(E)** KEGG analysis of module2 gene.

We used CytoNCA to score these 201 genes twice using multiple criteria (Figure 10A). Module one of 74 genes was obtained by the first calculation (Supplementary Table S2), and Module two of 36 genes was obtained by the second calculation (Supplementary Table S3). We took the second part of genes as core genes.

Next, functional enrichment analysis was performed on the genes of Module I and Module II (Figures 10B–E), kegg analysis of Module I gene showed that this part of gene was associated with hepatitis B, hepatitis C and HIF-1 pathway (Figure 10C), kegg analysis of module two genes suggested that these genes were associated with HIF-1 pathway and PD-L1 immunotherapy (Figure 10E), all of which are closely related to the occurrence and development of HCC.

## 4 Discussion

These findings indicate that the later the stage of HCC diagnosis and the younger the age of the patient, the faster the growth rate of HCC (Gao et al., 2021), TCM has been gaining increasing attention for the treatment of malignant tumors due to its low toxicity and high efficiency (Wang et al., 2020). Particularly in recent years, TCM has been widely utilized in the therapy and prevention of HCC and has been shown to be associated with various physiopathologic processes of HCC. Some studies have demonstrated that TCM not only has a preventive effect on HCC but also plays a role in inhibiting cell proliferation, disrupting the cell cycle, hindering epithelial-mesenchymal transition, and enhancing the effectiveness of cancer chemotherapy (Hu et al., 2016). TCM has amassed considerable expertise in the treatment of HCC, significantly contributing to improving clinical HCC symptoms, boosting immunity, enhancing survival rates, and improving the quality of life, among other benefits. Furthermore, TCM is characterized by its holistic regulation and multi-target intervention (Qi et al., 2015; Li K. et al., 2022). Although TCM had its natural advantages in disease management, it still had some drawbacks. Firstly, relative to Western medicine, the production, sale, and usage of TCM lacked standardization and regulation. Consequently, there might have been TCM products of varying quality and contaminated with harmful substances, making it difficult for patients to assess their safety and efficacy. Secondly, the dosage and composition of TCM were often not easily controllable, leading to unstable drug efficacy and increased side effects (Tu et al., 2021). This study’s meta-analysis can reconcile differences among various studies, address heterogeneity, employ network pharmacology to establish links between TCM’s multiple components and multiple targets.

The results of our meta-analysis reveal that combined TCM treatment can enhance the ORR and DCR of HCC patients compared to TACE and chemotherapy administered alone. Patients receiving TCM exhibited improved OS and RFS, although there was no significant impact on PFS and DFS. This lack of significance may be attributed to the limited number of studies reporting these indicators, potentially introducing bias, thus warranting further case reviews to validate these findings. In addition, HCC patients treated with TCM experienced effective reduction in gastrointestinal reactions. Studies have demonstrated that frequently used TCM components like Huangqi, Baishao, Chaihu, and Baizhu play a certain role in protecting the gastrointestinal mucosa and promoting peristalsis (Zhu et al., 2018; Huang et al., 2019; Zhou et al., 2019; Li et al., 2022). TCM also mitigates damage to the blood system in HCC patients, such as leukopenia and thrombocytopenia, with studies indicating that Huangqi can improve the function of hematopoietic organs like the thymus and spleen (Li et al., 2021). Most notably, HCC patients receiving combined TCM therapy encountered less liver damage. Studies suggest that Dangshen can alleviate liver inflammation and fibrosis processes (Hong et al., 2017), Chaihu has also been confirmed to improve the liver’s antioxidant capacity (Zhao et al., 2012), and animal models have demonstrated that Banzhilian provides a degree of protection against various forms of induced liver injury (Lin et al., 1994). These mechanisms collectively indicate that combined TCM therapy offers greater protection to the liver. The top five active ingredients identified in our screening are MOL000098, MOL000422, MOL000449, MOL000006, and MOL000354, corresponding to quercetin, kaempferol, stigmasterol, luteolin, and isorhamnetin. Studies have shown that quercetin is involved in HCC autophagy (Wu et al., 2022), kaempferol inhibits HCC cell migration (Ju et al., 2021), luteolin enhances the efficacy of chemotherapy drugs (Xu et al., 2016), and isorhamnetin prevents liver fibrosis through oxidative stress (Yang et al., 2016).

The functional enrichment analysis of the two modules revealed a common pathway enrichment associated with hepatitis B, Hepatitis C, the HIF-1 signaling pathway, and PD-L1-related pathways in cancer, these pathways were not only directly linked to HCC but also played roles in various aspects of HCC. Activation of the HIF-1 signaling pathway has been shown to promote HCC proliferation (Kung-Chun Chiu et al., 2019) and reduce HCC’s sensitivity to chemotherapy and radiotherapy (Zhao et al., 2014; Hu et al., 2021; Bai et al., 2022), PD-L1 and its related receptors have implications for HCC immunotherapy (Li et al., 2022; Qin, 2022). In these molecules, many were closely associated with HCC. For instance, the pathogenesis and epigenetics of HCC were closely related to TP53 (Hussain et al., 2007), the overexpression of the STAT3 molecule was associated with the poor prognosis of HCC (Lee and Cheung, 2019). *In vitro* models demonstrated that MYC played a direct role in inducing the transformation of liver cells into HCC cells (Zimonjic and Popescu, 2012), experimental evidence proved that the upregulation of MMP9 could promote the migration and invasion of HCC (Wang et al., 2016). HMOX1 altered the resistance of HCC to sorafenib by modulating the expression of ABC transporters (Zhu et al., 2022), The activation of the EGFR-STAT3-ABCB1 pathway was closely associated with chemotherapy drug resistance (Hu et al., 2022). Fos could promote the development of hepatocellular carcinoma by directly regulating the expression of The Brother of the Regulator of Imprinted Sites (BORIS) (Xian et al., 2024).

Nonetheless, this study had certain limitations. Firstly, the meta-analysis included 14 studies, and inherent factors like detection methods and follow-up durations may have introduced heterogeneity into the analysis. To mitigate this, future research will employ more stringent inclusion/exclusion criteria and emphasize subgroup analysis. Secondly, some studies lacked precise values when calculating 1-, 3- and 5-year survival rates, requiring us to extract data from the Kaplan-Meier curve using the tracking method, which may have introduced some errors. Finally, the distribution of the HCC gene of interest in both solid and peripheral tissues remained unclear in this study, necessitating further exploration.

This study is the first to integrate meta-analysis and network pharmacology to investigate the effectiveness of TCM against HCC and its potential pharmacological mechanisms. We hope that this study offers a fresh perspective on the clinical management of HCC and provides valuable insights and experimental directions for researchers in this field.

## 5 Conclusion

In summary, our study has revealed that the combination of TCM therapy for HCC is more effective and carries fewer side effects compared to monotherapy. Furthermore, the therapeutic impact of TCM on HCC is influenced by a multi-target, multi-component, and multi-pathway mechanism. To establish the reliability of these pathways, future research should involve more rigorous experimental design and necessitate further *in vivo* and *in vitro* pharmacological experiments for the validation of these mechanisms.

## Data Availability

The original contributions presented in the study are included in the article/Supplementary Material, further inquiries can be directed to the corresponding authors.

## Supplementary material

SUPPLEMENTARY FIGURE S1 Sensitivity analysis of other clinical outcome.

SUPPLEMENTARY FIGURE S2 Protein-protein interaction network of TCM-HCC targets.
