# Supplementary material for: Integration of meta-analysis and network pharmacology analysis to investigate the pharmacological mechanisms of traditional Chinese medicine in the treatment of hepatocellular carcinoma
Source: Front Pharmacol. 2024 Mar 15;15:1374988. doi: 10.3389/fphar.2024.1374988 (PMC10978761; doi:10.3389/fphar.2024.1374988)
Supplement: Supplementary file 1 [file Table1.DOCX]

TableS1: Summary of relevant information on pharmacological networks

| Node | Type | Baishao | Chaihu | Banzhilian | Baizhu | Danggui | Dangshen | Fuling | Gancao | Huangqi |
| --- | --- | --- | --- | --- | --- | --- | --- | --- | --- | --- |
| XIAP | gene | 0 | 0 | 0 | 0 | 0 | 0 | 0 | 0 | 0 |
| VEGFA | gene | 0 | 0 | 0 | 0 | 0 | 0 | 0 | 0 | 0 |
| VCAM1 | gene | 0 | 0 | 0 | 0 | 0 | 0 | 0 | 0 | 0 |
| UGT1A1 | gene | 0 | 0 | 0 | 0 | 0 | 0 | 0 | 0 | 0 |
| TYR | gene | 0 | 0 | 0 | 0 | 0 | 0 | 0 | 0 | 0 |
| TP53 | gene | 0 | 0 | 0 | 0 | 0 | 0 | 0 | 0 | 0 |
| TOP2A | gene | 0 | 0 | 0 | 0 | 0 | 0 | 0 | 0 | 0 |
| TOP1 | gene | 0 | 0 | 0 | 0 | 0 | 0 | 0 | 0 | 0 |
| THBD | gene | 0 | 0 | 0 | 0 | 0 | 0 | 0 | 0 | 0 |
| TEP1 | gene | 0 | 0 | 0 | 0 | 0 | 0 | 0 | 0 | 0 |
| SULT1E1 | gene | 0 | 0 | 0 | 0 | 0 | 0 | 0 | 0 | 0 |
| STAT3 | gene | 0 | 0 | 0 | 0 | 0 | 0 | 0 | 0 | 0 |
| STAT1 | gene | 0 | 0 | 0 | 0 | 0 | 0 | 0 | 0 | 0 |
| SREBF1 | gene | 0 | 0 | 0 | 0 | 0 | 0 | 0 | 0 | 0 |
| SPP1 | gene | 0 | 0 | 0 | 0 | 0 | 0 | 0 | 0 | 0 |
| SOD1 | gene | 0 | 0 | 0 | 0 | 0 | 0 | 0 | 0 | 0 |
| SOAT2 | gene | 0 | 0 | 0 | 0 | 0 | 0 | 0 | 0 | 0 |
| SOAT1 | gene | 0 | 0 | 0 | 0 | 0 | 0 | 0 | 0 | 0 |
| SLPI | gene | 0 | 0 | 0 | 0 | 0 | 0 | 0 | 0 | 0 |
| SLC6A2 | gene | 0 | 0 | 0 | 0 | 0 | 0 | 0 | 0 | 0 |
| SLC2A4 | gene | 0 | 0 | 0 | 0 | 0 | 0 | 0 | 0 | 0 |
| SERPINE1 | gene | 0 | 0 | 0 | 0 | 0 | 0 | 0 | 0 | 0 |
| SELE | gene | 0 | 0 | 0 | 0 | 0 | 0 | 0 | 0 | 0 |
| RXRB | gene | 0 | 0 | 0 | 0 | 0 | 0 | 0 | 0 | 0 |
| RXRA | gene | 0 | 0 | 0 | 0 | 0 | 0 | 0 | 0 | 0 |
| RUNX2 | gene | 0 | 0 | 0 | 0 | 0 | 0 | 0 | 0 | 0 |
| RELA | gene | 0 | 0 | 0 | 0 | 0 | 0 | 0 | 0 | 0 |
| RB1 | gene | 0 | 0 | 0 | 0 | 0 | 0 | 0 | 0 | 0 |
| RASSF1 | gene | 0 | 0 | 0 | 0 | 0 | 0 | 0 | 0 | 0 |
| RASA1 | gene | 0 | 0 | 0 | 0 | 0 | 0 | 0 | 0 | 0 |
| RAF1 | gene | 0 | 0 | 0 | 0 | 0 | 0 | 0 | 0 | 0 |
| PYGM | gene | 0 | 0 | 0 | 0 | 0 | 0 | 0 | 0 | 0 |
| PTGS2 | gene | 0 | 0 | 0 | 0 | 0 | 0 | 0 | 0 | 0 |
| PTGS1 | gene | 0 | 0 | 0 | 0 | 0 | 0 | 0 | 0 | 0 |
| PTGES | gene | 0 | 0 | 0 | 0 | 0 | 0 | 0 | 0 | 0 |
| PSMD3 | gene | 0 | 0 | 0 | 0 | 0 | 0 | 0 | 0 | 0 |
| PRSS1 | gene | 0 | 0 | 0 | 0 | 0 | 0 | 0 | 0 | 0 |
| PRKCD | gene | 0 | 0 | 0 | 0 | 0 | 0 | 0 | 0 | 0 |
| PRKCB | gene | 0 | 0 | 0 | 0 | 0 | 0 | 0 | 0 | 0 |
| PRKCA | gene | 0 | 0 | 0 | 0 | 0 | 0 | 0 | 0 | 0 |
| PPARG | gene | 0 | 0 | 0 | 0 | 0 | 0 | 0 | 0 | 0 |
| PPARD | gene | 0 | 0 | 0 | 0 | 0 | 0 | 0 | 0 | 0 |
| PPARA | gene | 0 | 0 | 0 | 0 | 0 | 0 | 0 | 0 | 0 |
| POR | gene | 0 | 0 | 0 | 0 | 0 | 0 | 0 | 0 | 0 |
| PON1 | gene | 0 | 0 | 0 | 0 | 0 | 0 | 0 | 0 | 0 |
| PLB1 | gene | 0 | 0 | 0 | 0 | 0 | 0 | 0 | 0 | 0 |
| PLAU | gene | 0 | 0 | 0 | 0 | 0 | 0 | 0 | 0 | 0 |
| PLAT | gene | 0 | 0 | 0 | 0 | 0 | 0 | 0 | 0 | 0 |
| PGR | gene | 0 | 0 | 0 | 0 | 0 | 0 | 0 | 0 | 0 |
| PCNA | gene | 0 | 0 | 0 | 0 | 0 | 0 | 0 | 0 | 0 |
| PARP1 | gene | 0 | 0 | 0 | 0 | 0 | 0 | 0 | 0 | 0 |
| OPRM1 | gene | 0 | 0 | 0 | 0 | 0 | 0 | 0 | 0 | 0 |
| ODC1 | gene | 0 | 0 | 0 | 0 | 0 | 0 | 0 | 0 | 0 |
| NUF2 | gene | 0 | 0 | 0 | 0 | 0 | 0 | 0 | 0 | 0 |
| NR3C2 | gene | 0 | 0 | 0 | 0 | 0 | 0 | 0 | 0 | 0 |
| NR3C1 | gene | 0 | 0 | 0 | 0 | 0 | 0 | 0 | 0 | 0 |
| NR1I3 | gene | 0 | 0 | 0 | 0 | 0 | 0 | 0 | 0 | 0 |
| NR1I2 | gene | 0 | 0 | 0 | 0 | 0 | 0 | 0 | 0 | 0 |
| NQO1 | gene | 0 | 0 | 0 | 0 | 0 | 0 | 0 | 0 | 0 |
| NPEPPS | gene | 0 | 0 | 0 | 0 | 0 | 0 | 0 | 0 | 0 |
| NOS3 | gene | 0 | 0 | 0 | 0 | 0 | 0 | 0 | 0 | 0 |
| NOS2 | gene | 0 | 0 | 0 | 0 | 0 | 0 | 0 | 0 | 0 |
| NFKBIA | gene | 0 | 0 | 0 | 0 | 0 | 0 | 0 | 0 | 0 |
| NFE2L2 | gene | 0 | 0 | 0 | 0 | 0 | 0 | 0 | 0 | 0 |
| NFATC1 | gene | 0 | 0 | 0 | 0 | 0 | 0 | 0 | 0 | 0 |
| NCOA2 | gene | 0 | 0 | 0 | 0 | 0 | 0 | 0 | 0 | 0 |
| NCOA1 | gene | 0 | 0 | 0 | 0 | 0 | 0 | 0 | 0 | 0 |
| MYC | gene | 0 | 0 | 0 | 0 | 0 | 0 | 0 | 0 | 0 |
| MTTP | gene | 0 | 0 | 0 | 0 | 0 | 0 | 0 | 0 | 0 |
| MPO | gene | 0 | 0 | 0 | 0 | 0 | 0 | 0 | 0 | 0 |
| MMP9 | gene | 0 | 0 | 0 | 0 | 0 | 0 | 0 | 0 | 0 |
| MMP8 | gene | 0 | 0 | 0 | 0 | 0 | 0 | 0 | 0 | 0 |
| MMP3 | gene | 0 | 0 | 0 | 0 | 0 | 0 | 0 | 0 | 0 |
| MMP2 | gene | 0 | 0 | 0 | 0 | 0 | 0 | 0 | 0 | 0 |
| MMP13 | gene | 0 | 0 | 0 | 0 | 0 | 0 | 0 | 0 | 0 |
| MMP1 | gene | 0 | 0 | 0 | 0 | 0 | 0 | 0 | 0 | 0 |
| MET | gene | 0 | 0 | 0 | 0 | 0 | 0 | 0 | 0 | 0 |
| MDM2 | gene | 0 | 0 | 0 | 0 | 0 | 0 | 0 | 0 | 0 |
| MCL1 | gene | 0 | 0 | 0 | 0 | 0 | 0 | 0 | 0 | 0 |
| MAPK8 | gene | 0 | 0 | 0 | 0 | 0 | 0 | 0 | 0 | 0 |
| MAPK3 | gene | 0 | 0 | 0 | 0 | 0 | 0 | 0 | 0 | 0 |
| MAPK14 | gene | 0 | 0 | 0 | 0 | 0 | 0 | 0 | 0 | 0 |
| MAPK10 | gene | 0 | 0 | 0 | 0 | 0 | 0 | 0 | 0 | 0 |
| MAPK1 | gene | 0 | 0 | 0 | 0 | 0 | 0 | 0 | 0 | 0 |
| MAP2 | gene | 0 | 0 | 0 | 0 | 0 | 0 | 0 | 0 | 0 |
| MAOA | gene | 0 | 0 | 0 | 0 | 0 | 0 | 0 | 0 | 0 |
| LYZ | gene | 0 | 0 | 0 | 0 | 0 | 0 | 0 | 0 | 0 |
| LTA4H | gene | 0 | 0 | 0 | 0 | 0 | 0 | 0 | 0 | 0 |
| LDLR | gene | 0 | 0 | 0 | 0 | 0 | 0 | 0 | 0 | 0 |
| KDR | gene | 0 | 0 | 0 | 0 | 0 | 0 | 0 | 0 | 0 |
| IRF1 | gene | 0 | 0 | 0 | 0 | 0 | 0 | 0 | 0 | 0 |
| INSR | gene | 0 | 0 | 0 | 0 | 0 | 0 | 0 | 0 | 0 |
| IL6ST | gene | 0 | 0 | 0 | 0 | 0 | 0 | 0 | 0 | 0 |
| IL4 | gene | 0 | 0 | 0 | 0 | 0 | 0 | 0 | 0 | 0 |
| IL2RA | gene | 0 | 0 | 0 | 0 | 0 | 0 | 0 | 0 | 0 |
| IL1B | gene | 0 | 0 | 0 | 0 | 0 | 0 | 0 | 0 | 0 |
| IL1A | gene | 0 | 0 | 0 | 0 | 0 | 0 | 0 | 0 | 0 |
| IL10 | gene | 0 | 0 | 0 | 0 | 0 | 0 | 0 | 0 | 0 |
| IKBKB | gene | 0 | 0 | 0 | 0 | 0 | 0 | 0 | 0 | 0 |
| IGFBP3 | gene | 0 | 0 | 0 | 0 | 0 | 0 | 0 | 0 | 0 |
| IGF2 | gene | 0 | 0 | 0 | 0 | 0 | 0 | 0 | 0 | 0 |
| IFNG | gene | 0 | 0 | 0 | 0 | 0 | 0 | 0 | 0 | 0 |
| ICAM1 | gene | 0 | 0 | 0 | 0 | 0 | 0 | 0 | 0 | 0 |
| HSPB1 | gene | 0 | 0 | 0 | 0 | 0 | 0 | 0 | 0 | 0 |
| HSPA5 | gene | 0 | 0 | 0 | 0 | 0 | 0 | 0 | 0 | 0 |
| HSF1 | gene | 0 | 0 | 0 | 0 | 0 | 0 | 0 | 0 | 0 |
| HSD3B2 | gene | 0 | 0 | 0 | 0 | 0 | 0 | 0 | 0 | 0 |
| HMOX1 | gene | 0 | 0 | 0 | 0 | 0 | 0 | 0 | 0 | 0 |
| HMGCR | gene | 0 | 0 | 0 | 0 | 0 | 0 | 0 | 0 | 0 |
| HK2 | gene | 0 | 0 | 0 | 0 | 0 | 0 | 0 | 0 | 0 |
| HIF1A | gene | 0 | 0 | 0 | 0 | 0 | 0 | 0 | 0 | 0 |
| GSTP1 | gene | 0 | 0 | 0 | 0 | 0 | 0 | 0 | 0 | 0 |
| GSTM2 | gene | 0 | 0 | 0 | 0 | 0 | 0 | 0 | 0 | 0 |
| GSTM1 | gene | 0 | 0 | 0 | 0 | 0 | 0 | 0 | 0 | 0 |
| GSR | gene | 0 | 0 | 0 | 0 | 0 | 0 | 0 | 0 | 0 |
| GSK3B | gene | 0 | 0 | 0 | 0 | 0 | 0 | 0 | 0 | 0 |
| GOT1 | gene | 0 | 0 | 0 | 0 | 0 | 0 | 0 | 0 | 0 |
| GJA1 | gene | 0 | 0 | 0 | 0 | 0 | 0 | 0 | 0 | 0 |
| FOSL2 | gene | 0 | 0 | 0 | 0 | 0 | 0 | 0 | 0 | 0 |
| FOSL1 | gene | 0 | 0 | 0 | 0 | 0 | 0 | 0 | 0 | 0 |
| FOS | gene | 0 | 0 | 0 | 0 | 0 | 0 | 0 | 0 | 0 |
| FN1 | gene | 0 | 0 | 0 | 0 | 0 | 0 | 0 | 0 | 0 |
| FASN | gene | 0 | 0 | 0 | 0 | 0 | 0 | 0 | 0 | 0 |
| FABP5 | gene | 0 | 0 | 0 | 0 | 0 | 0 | 0 | 0 | 0 |
| F7 | gene | 0 | 0 | 0 | 0 | 0 | 0 | 0 | 0 | 0 |
| F3 | gene | 0 | 0 | 0 | 0 | 0 | 0 | 0 | 0 | 0 |
| ESR2 | gene | 0 | 0 | 0 | 0 | 0 | 0 | 0 | 0 | 0 |
| ESR1 | gene | 0 | 0 | 0 | 0 | 0 | 0 | 0 | 0 | 0 |
| ERBB3 | gene | 0 | 0 | 0 | 0 | 0 | 0 | 0 | 0 | 0 |
| ERBB2 | gene | 0 | 0 | 0 | 0 | 0 | 0 | 0 | 0 | 0 |
| ELK1 | gene | 0 | 0 | 0 | 0 | 0 | 0 | 0 | 0 | 0 |
| EIF6 | gene | 0 | 0 | 0 | 0 | 0 | 0 | 0 | 0 | 0 |
| EGLN1 | gene | 0 | 0 | 0 | 0 | 0 | 0 | 0 | 0 | 0 |
| EGFR | gene | 0 | 0 | 0 | 0 | 0 | 0 | 0 | 0 | 0 |
| EGF | gene | 0 | 0 | 0 | 0 | 0 | 0 | 0 | 0 | 0 |
| E2F2 | gene | 0 | 0 | 0 | 0 | 0 | 0 | 0 | 0 | 0 |
| E2F1 | gene | 0 | 0 | 0 | 0 | 0 | 0 | 0 | 0 | 0 |
| DUOX2 | gene | 0 | 0 | 0 | 0 | 0 | 0 | 0 | 0 | 0 |
| DPP4 | gene | 0 | 0 | 0 | 0 | 0 | 0 | 0 | 0 | 0 |
| CYP3A4 | gene | 0 | 0 | 0 | 0 | 0 | 0 | 0 | 0 | 0 |
| CYP1B1 | gene | 0 | 0 | 0 | 0 | 0 | 0 | 0 | 0 | 0 |
| CYP1A2 | gene | 0 | 0 | 0 | 0 | 0 | 0 | 0 | 0 | 0 |
| CYP1A1 | gene | 0 | 0 | 0 | 0 | 0 | 0 | 0 | 0 | 0 |
| CYP19A1 | gene | 0 | 0 | 0 | 0 | 0 | 0 | 0 | 0 | 0 |
| CYCS | gene | 0 | 0 | 0 | 0 | 0 | 0 | 0 | 0 | 0 |
| CXCL8 | gene | 0 | 0 | 0 | 0 | 0 | 0 | 0 | 0 | 0 |
| CXCL2 | gene | 0 | 0 | 0 | 0 | 0 | 0 | 0 | 0 | 0 |
| CXCL11 | gene | 0 | 0 | 0 | 0 | 0 | 0 | 0 | 0 | 0 |
| CXCL10 | gene | 0 | 0 | 0 | 0 | 0 | 0 | 0 | 0 | 0 |
| CTSD | gene | 0 | 0 | 0 | 0 | 0 | 0 | 0 | 0 | 0 |
| COL3A1 | gene | 0 | 0 | 0 | 0 | 0 | 0 | 0 | 0 | 0 |
| COL1A1 | gene | 0 | 0 | 0 | 0 | 0 | 0 | 0 | 0 | 0 |
| CLDN4 | gene | 0 | 0 | 0 | 0 | 0 | 0 | 0 | 0 | 0 |
| CHUK | gene | 0 | 0 | 0 | 0 | 0 | 0 | 0 | 0 | 0 |
| CHRM3 | gene | 0 | 0 | 0 | 0 | 0 | 0 | 0 | 0 | 0 |
| CHEK2 | gene | 0 | 0 | 0 | 0 | 0 | 0 | 0 | 0 | 0 |
| CHEK1 | gene | 0 | 0 | 0 | 0 | 0 | 0 | 0 | 0 | 0 |
| CES1 | gene | 0 | 0 | 0 | 0 | 0 | 0 | 0 | 0 | 0 |
| CDKN1A | gene | 0 | 0 | 0 | 0 | 0 | 0 | 0 | 0 | 0 |
| CDK4 | gene | 0 | 0 | 0 | 0 | 0 | 0 | 0 | 0 | 0 |
| CDK2 | gene | 0 | 0 | 0 | 0 | 0 | 0 | 0 | 0 | 0 |
| CDK1 | gene | 0 | 0 | 0 | 0 | 0 | 0 | 0 | 0 | 0 |
| CD40LG | gene | 0 | 0 | 0 | 0 | 0 | 0 | 0 | 0 | 0 |
| CD14 | gene | 0 | 0 | 0 | 0 | 0 | 0 | 0 | 0 | 0 |
| CCND1 | gene | 0 | 0 | 0 | 0 | 0 | 0 | 0 | 0 | 0 |
| CCNB1 | gene | 0 | 0 | 0 | 0 | 0 | 0 | 0 | 0 | 0 |
| CCNA2 | gene | 0 | 0 | 0 | 0 | 0 | 0 | 0 | 0 | 0 |
| CCL2 | gene | 0 | 0 | 0 | 0 | 0 | 0 | 0 | 0 | 0 |
| CAV1 | gene | 0 | 0 | 0 | 0 | 0 | 0 | 0 | 0 | 0 |
| CAT | gene | 0 | 0 | 0 | 0 | 0 | 0 | 0 | 0 | 0 |
| CASP9 | gene | 0 | 0 | 0 | 0 | 0 | 0 | 0 | 0 | 0 |
| CASP8 | gene | 0 | 0 | 0 | 0 | 0 | 0 | 0 | 0 | 0 |
| CASP7 | gene | 0 | 0 | 0 | 0 | 0 | 0 | 0 | 0 | 0 |
| CASP3 | gene | 0 | 0 | 0 | 0 | 0 | 0 | 0 | 0 | 0 |
| CA2 | gene | 0 | 0 | 0 | 0 | 0 | 0 | 0 | 0 | 0 |
| BIRC5 | gene | 0 | 0 | 0 | 0 | 0 | 0 | 0 | 0 | 0 |
| BCL2L1 | gene | 0 | 0 | 0 | 0 | 0 | 0 | 0 | 0 | 0 |
| BCL2 | gene | 0 | 0 | 0 | 0 | 0 | 0 | 0 | 0 | 0 |
| BAX | gene | 0 | 0 | 0 | 0 | 0 | 0 | 0 | 0 | 0 |
| BAD | gene | 0 | 0 | 0 | 0 | 0 | 0 | 0 | 0 | 0 |
| ATP5F1B | gene | 0 | 0 | 0 | 0 | 0 | 0 | 0 | 0 | 0 |
| AR | gene | 0 | 0 | 0 | 0 | 0 | 0 | 0 | 0 | 0 |
| APP | gene | 0 | 0 | 0 | 0 | 0 | 0 | 0 | 0 | 0 |
| APOD | gene | 0 | 0 | 0 | 0 | 0 | 0 | 0 | 0 | 0 |
| APOB | gene | 0 | 0 | 0 | 0 | 0 | 0 | 0 | 0 | 0 |
| ALOX5 | gene | 0 | 0 | 0 | 0 | 0 | 0 | 0 | 0 | 0 |
| AKT1 | gene | 0 | 0 | 0 | 0 | 0 | 0 | 0 | 0 | 0 |
| AKR1C3 | gene | 0 | 0 | 0 | 0 | 0 | 0 | 0 | 0 | 0 |
| AKR1C1 | gene | 0 | 0 | 0 | 0 | 0 | 0 | 0 | 0 | 0 |
| AKR1B1 | gene | 0 | 0 | 0 | 0 | 0 | 0 | 0 | 0 | 0 |
| AHSA1 | gene | 0 | 0 | 0 | 0 | 0 | 0 | 0 | 0 | 0 |
| AHR | gene | 0 | 0 | 0 | 0 | 0 | 0 | 0 | 0 | 0 |
| ADRB2 | gene | 0 | 0 | 0 | 0 | 0 | 0 | 0 | 0 | 0 |
| ADRA1B | gene | 0 | 0 | 0 | 0 | 0 | 0 | 0 | 0 | 0 |
| ADRA1A | gene | 0 | 0 | 0 | 0 | 0 | 0 | 0 | 0 | 0 |
| ADIPOR2 | gene | 0 | 0 | 0 | 0 | 0 | 0 | 0 | 0 | 0 |
| ADH1C | gene | 0 | 0 | 0 | 0 | 0 | 0 | 0 | 0 | 0 |
| ADH1B | gene | 0 | 0 | 0 | 0 | 0 | 0 | 0 | 0 | 0 |
| ACACA | gene | 0 | 0 | 0 | 0 | 0 | 0 | 0 | 0 | 0 |
| ABCG2 | gene | 0 | 0 | 0 | 0 | 0 | 0 | 0 | 0 | 0 |
| ABCC1 | gene | 0 | 0 | 0 | 0 | 0 | 0 | 0 | 0 | 0 |
| MOL004806 | Gancao | 0 | 0 | 0 | 0 | 0 | 0 | 0 | 1 | 0 |
| MOL000033 | multiDrug | 0 | 0 | 0 | 1 | 0 | 0 | 0 | 0 | 1 |
| MOL000211 | multiDrug | 1 | 0 | 0 | 0 | 0 | 0 | 0 | 1 | 1 |
| MOL004985 | Gancao | 0 | 0 | 0 | 0 | 0 | 0 | 0 | 1 | 0 |
| MOL000239 | multiDrug | 0 | 0 | 0 | 0 | 0 | 0 | 0 | 1 | 1 |
| MOL004328 | Gancao | 0 | 0 | 0 | 0 | 0 | 0 | 0 | 1 | 0 |
| MOL000275 | Fuling | 0 | 0 | 0 | 0 | 0 | 0 | 1 | 0 | 0 |
| MOL000173 | Banzhilian | 0 | 0 | 1 | 0 | 0 | 0 | 0 | 0 | 0 |
| MOL006774 | Dangshen | 0 | 0 | 0 | 0 | 0 | 1 | 0 | 0 | 0 |
| MOL012254 | Banzhilian | 0 | 0 | 1 | 0 | 0 | 0 | 0 | 0 | 0 |
| MOL004980 | Gancao | 0 | 0 | 0 | 0 | 0 | 0 | 0 | 1 | 0 |
| MOL004864 | Gancao | 0 | 0 | 0 | 0 | 0 | 0 | 0 | 1 | 0 |
| MOL004808 | Gancao | 0 | 0 | 0 | 0 | 0 | 0 | 0 | 1 | 0 |
| MOL000490 | Chaihu | 0 | 1 | 0 | 0 | 0 | 0 | 0 | 0 | 0 |
| MOL004924 | Gancao | 0 | 0 | 0 | 0 | 0 | 0 | 0 | 1 | 0 |
| MOL000072 | Baizhu | 0 | 0 | 0 | 1 | 0 | 0 | 0 | 0 | 0 |
| MOL004829 | Gancao | 0 | 0 | 0 | 0 | 0 | 0 | 0 | 1 | 0 |
| MOL004718 | Chaihu | 0 | 1 | 0 | 0 | 0 | 0 | 0 | 0 | 0 |
| MOL000492 | Baishao | 1 | 0 | 0 | 0 | 0 | 0 | 0 | 0 | 0 |
| MOL004598 | Chaihu | 0 | 1 | 0 | 0 | 0 | 0 | 0 | 0 | 0 |
| MOL008400 | Dangshen | 0 | 0 | 0 | 0 | 0 | 1 | 0 | 0 | 0 |
| MOL012251 | Banzhilian | 0 | 0 | 1 | 0 | 0 | 0 | 0 | 0 | 0 |
| MOL005190 | Banzhilian | 0 | 0 | 1 | 0 | 0 | 0 | 0 | 0 | 0 |
| MOL004820 | Gancao | 0 | 0 | 0 | 0 | 0 | 0 | 0 | 1 | 0 |
| MOL004866 | Gancao | 0 | 0 | 0 | 0 | 0 | 0 | 0 | 1 | 0 |
| MOL004857 | Gancao | 0 | 0 | 0 | 0 | 0 | 0 | 0 | 1 | 0 |
| MOL012245 | Banzhilian | 0 | 0 | 1 | 0 | 0 | 0 | 0 | 0 | 0 |
| MOL002311 | Gancao | 0 | 0 | 0 | 0 | 0 | 0 | 0 | 1 | 0 |
| MOL000433 | Huangqi | 0 | 0 | 0 | 0 | 0 | 0 | 0 | 0 | 1 |
| MOL005869 | Banzhilian | 0 | 0 | 1 | 0 | 0 | 0 | 0 | 0 | 0 |
| MOL004903 | Gancao | 0 | 0 | 0 | 0 | 0 | 0 | 0 | 1 | 0 |
| MOL005016 | Gancao | 0 | 0 | 0 | 0 | 0 | 0 | 0 | 1 | 0 |
| MOL005018 | Gancao | 0 | 0 | 0 | 0 | 0 | 0 | 0 | 1 | 0 |
| MOL000273 | Fuling | 0 | 0 | 0 | 0 | 0 | 0 | 1 | 0 | 0 |
| MOL000279 | Fuling | 0 | 0 | 0 | 0 | 0 | 0 | 1 | 0 | 0 |
| MOL004884 | Gancao | 0 | 0 | 0 | 0 | 0 | 0 | 0 | 1 | 0 |
| MOL004989 | Gancao | 0 | 0 | 0 | 0 | 0 | 0 | 0 | 1 | 0 |
| MOL012246 | Banzhilian | 0 | 0 | 1 | 0 | 0 | 0 | 0 | 0 | 0 |
| MOL005012 | Gancao | 0 | 0 | 0 | 0 | 0 | 0 | 0 | 1 | 0 |
| MOL000098 | multiDrug | 0 | 1 | 1 | 0 | 0 | 0 | 0 | 1 | 1 |
| MOL004828 | Gancao | 0 | 0 | 0 | 0 | 0 | 0 | 0 | 1 | 0 |
| MOL002915 | Banzhilian | 0 | 0 | 1 | 0 | 0 | 0 | 0 | 0 | 0 |
| MOL001755 | Banzhilian | 0 | 0 | 1 | 0 | 0 | 0 | 0 | 0 | 0 |
| MOL004883 | Gancao | 0 | 0 | 0 | 0 | 0 | 0 | 0 | 1 | 0 |
| MOL004624 | Chaihu | 0 | 1 | 0 | 0 | 0 | 0 | 0 | 0 | 0 |
| MOL000442 | Huangqi | 0 | 0 | 0 | 0 | 0 | 0 | 0 | 0 | 1 |
| MOL000296 | multiDrug | 0 | 0 | 0 | 0 | 0 | 0 | 1 | 0 | 1 |
| MOL012248 | Banzhilian | 0 | 0 | 1 | 0 | 0 | 0 | 0 | 0 | 0 |
| MOL012250 | Banzhilian | 0 | 0 | 1 | 0 | 0 | 0 | 0 | 0 | 0 |
| MOL000049 | Baizhu | 0 | 0 | 0 | 1 | 0 | 0 | 0 | 0 | 0 |
| MOL012252 | Banzhilian | 0 | 0 | 1 | 0 | 0 | 0 | 0 | 0 | 0 |
| MOL004957 | Gancao | 0 | 0 | 0 | 0 | 0 | 0 | 0 | 1 | 0 |
| MOL002879 | Dangshen | 0 | 0 | 0 | 0 | 0 | 1 | 0 | 0 | 0 |
| MOL004966 | Gancao | 0 | 0 | 0 | 0 | 0 | 0 | 0 | 1 | 0 |
| MOL001040 | Banzhilian | 0 | 0 | 1 | 0 | 0 | 0 | 0 | 0 | 0 |
| MOL012270 | Banzhilian | 0 | 0 | 1 | 0 | 0 | 0 | 0 | 0 | 0 |
| MOL004904 | Gancao | 0 | 0 | 0 | 0 | 0 | 0 | 0 | 1 | 0 |
| MOL005007 | Gancao | 0 | 0 | 0 | 0 | 0 | 0 | 0 | 1 | 0 |
| MOL003036 | Dangshen | 0 | 0 | 0 | 0 | 0 | 1 | 0 | 0 | 0 |
| MOL008206 | Banzhilian | 0 | 0 | 1 | 0 | 0 | 0 | 0 | 0 | 0 |
| MOL001735 | Banzhilian | 0 | 0 | 1 | 0 | 0 | 0 | 0 | 0 | 0 |
| MOL004805 | Gancao | 0 | 0 | 0 | 0 | 0 | 0 | 0 | 1 | 0 |
| MOL001792 | Gancao | 0 | 0 | 0 | 0 | 0 | 0 | 0 | 1 | 0 |
| MOL000449 | multiDrug | 0 | 1 | 1 | 0 | 1 | 1 | 0 | 0 | 0 |
| MOL005321 | Dangshen | 0 | 0 | 0 | 0 | 0 | 1 | 0 | 0 | 0 |
| MOL004882 | Gancao | 0 | 0 | 0 | 0 | 0 | 0 | 0 | 1 | 0 |
| MOL004824 | Gancao | 0 | 0 | 0 | 0 | 0 | 0 | 0 | 1 | 0 |
| MOL000380 | Huangqi | 0 | 0 | 0 | 0 | 0 | 0 | 0 | 0 | 1 |
| MOL004988 | Gancao | 0 | 0 | 0 | 0 | 0 | 0 | 0 | 1 | 0 |
| MOL001924 | Baishao | 1 | 0 | 0 | 0 | 0 | 0 | 0 | 0 | 0 |
| MOL004961 | Gancao | 0 | 0 | 0 | 0 | 0 | 0 | 0 | 1 | 0 |
| MOL004885 | Gancao | 0 | 0 | 0 | 0 | 0 | 0 | 0 | 1 | 0 |
| MOL004609 | Chaihu | 0 | 1 | 0 | 0 | 0 | 0 | 0 | 0 | 0 |
| MOL004863 | Gancao | 0 | 0 | 0 | 0 | 0 | 0 | 0 | 1 | 0 |
| MOL004908 | Gancao | 0 | 0 | 0 | 0 | 0 | 0 | 0 | 1 | 0 |
| MOL002565 | Gancao | 0 | 0 | 0 | 0 | 0 | 0 | 0 | 1 | 0 |
| MOL004935 | Gancao | 0 | 0 | 0 | 0 | 0 | 0 | 0 | 1 | 0 |
| MOL000283 | Fuling | 0 | 0 | 0 | 0 | 0 | 0 | 1 | 0 | 0 |
| MOL004849 | Gancao | 0 | 0 | 0 | 0 | 0 | 0 | 0 | 1 | 0 |
| MOL000354 | multiDrug | 0 | 1 | 0 | 0 | 0 | 0 | 0 | 1 | 1 |
| MOL004898 | Gancao | 0 | 0 | 0 | 0 | 0 | 0 | 0 | 1 | 0 |
| MOL000371 | Huangqi | 0 | 0 | 0 | 0 | 0 | 0 | 0 | 0 | 1 |
| MOL000417 | multiDrug | 0 | 0 | 0 | 0 | 0 | 0 | 0 | 1 | 1 |
| MOL000387 | Huangqi | 0 | 0 | 0 | 0 | 0 | 0 | 0 | 0 | 1 |
| MOL004959 | Gancao | 0 | 0 | 0 | 0 | 0 | 0 | 0 | 1 | 0 |
| MOL002714 | Banzhilian | 0 | 0 | 1 | 0 | 0 | 0 | 0 | 0 | 0 |
| MOL004815 | Gancao | 0 | 0 | 0 | 0 | 0 | 0 | 0 | 1 | 0 |
| MOL004941 | Gancao | 0 | 0 | 0 | 0 | 0 | 0 | 0 | 1 | 0 |
| MOL008397 | Dangshen | 0 | 0 | 0 | 0 | 0 | 1 | 0 | 0 | 0 |
| MOL004990 | Gancao | 0 | 0 | 0 | 0 | 0 | 0 | 0 | 1 | 0 |
| MOL004810 | Gancao | 0 | 0 | 0 | 0 | 0 | 0 | 0 | 1 | 0 |
| MOL000351 | Banzhilian | 0 | 0 | 1 | 0 | 0 | 0 | 0 | 0 | 0 |
| MOL005003 | Gancao | 0 | 0 | 0 | 0 | 0 | 0 | 0 | 1 | 0 |
| MOL004355 | Dangshen | 0 | 0 | 0 | 0 | 0 | 1 | 0 | 0 | 0 |
| MOL004838 | Gancao | 0 | 0 | 0 | 0 | 0 | 0 | 0 | 1 | 0 |
| MOL001919 | Baishao | 1 | 0 | 0 | 0 | 0 | 0 | 0 | 0 | 0 |
| MOL004948 | Gancao | 0 | 0 | 0 | 0 | 0 | 0 | 0 | 1 | 0 |
| MOL005017 | Gancao | 0 | 0 | 0 | 0 | 0 | 0 | 0 | 1 | 0 |
| MOL004914 | Gancao | 0 | 0 | 0 | 0 | 0 | 0 | 0 | 1 | 0 |
| MOL000358 | multiDrug | 1 | 0 | 1 | 0 | 1 | 0 | 0 | 0 | 0 |
| MOL000500 | Gancao | 0 | 0 | 0 | 0 | 0 | 0 | 0 | 1 | 0 |
| MOL000282 | Fuling | 0 | 0 | 0 | 0 | 0 | 0 | 1 | 0 | 0 |
| MOL004993 | Gancao | 0 | 0 | 0 | 0 | 0 | 0 | 0 | 1 | 0 |
| MOL005000 | Gancao | 0 | 0 | 0 | 0 | 0 | 0 | 0 | 1 | 0 |
| MOL002140 | Dangshen | 0 | 0 | 0 | 0 | 0 | 1 | 0 | 0 | 0 |
| MOL004911 | Gancao | 0 | 0 | 0 | 0 | 0 | 0 | 0 | 1 | 0 |
| MOL004891 | Gancao | 0 | 0 | 0 | 0 | 0 | 0 | 0 | 1 | 0 |
| MOL004856 | Gancao | 0 | 0 | 0 | 0 | 0 | 0 | 0 | 1 | 0 |
| MOL000378 | Huangqi | 0 | 0 | 0 | 0 | 0 | 0 | 0 | 0 | 1 |
| MOL012266 | Banzhilian | 0 | 0 | 1 | 0 | 0 | 0 | 0 | 0 | 0 |
| MOL008411 | Dangshen | 0 | 0 | 0 | 0 | 0 | 1 | 0 | 0 | 0 |
| MOL000953 | Banzhilian | 0 | 0 | 1 | 0 | 0 | 0 | 0 | 0 | 0 |
| MOL004833 | Gancao | 0 | 0 | 0 | 0 | 0 | 0 | 0 | 1 | 0 |
| MOL005008 | Gancao | 0 | 0 | 0 | 0 | 0 | 0 | 0 | 1 | 0 |
| MOL000006 | multiDrug | 0 | 0 | 1 | 0 | 0 | 1 | 0 | 0 | 0 |
| MOL003656 | Gancao | 0 | 0 | 0 | 0 | 0 | 0 | 0 | 1 | 0 |
| MOL004991 | Gancao | 0 | 0 | 0 | 0 | 0 | 0 | 0 | 1 | 0 |
| MOL004653 | Chaihu | 0 | 1 | 0 | 0 | 0 | 0 | 0 | 0 | 0 |
| MOL004811 | Gancao | 0 | 0 | 0 | 0 | 0 | 0 | 0 | 1 | 0 |
| MOL004945 | Gancao | 0 | 0 | 0 | 0 | 0 | 0 | 0 | 1 | 0 |
| MOL007059 | Dangshen | 0 | 0 | 0 | 0 | 0 | 1 | 0 | 0 | 0 |
| MOL004848 | Gancao | 0 | 0 | 0 | 0 | 0 | 0 | 0 | 1 | 0 |
| MOL004978 | Gancao | 0 | 0 | 0 | 0 | 0 | 0 | 0 | 1 | 0 |
| MOL004827 | Gancao | 0 | 0 | 0 | 0 | 0 | 0 | 0 | 1 | 0 |
| MOL003896 | multiDrug | 0 | 0 | 0 | 0 | 0 | 1 | 0 | 1 | 0 |
| MOL000422 | multiDrug | 1 | 1 | 0 | 0 | 0 | 0 | 0 | 1 | 1 |
| MOL004913 | Gancao | 0 | 0 | 0 | 0 | 0 | 0 | 0 | 1 | 0 |
| MOL004855 | Gancao | 0 | 0 | 0 | 0 | 0 | 0 | 0 | 1 | 0 |
| MOL004814 | Gancao | 0 | 0 | 0 | 0 | 0 | 0 | 0 | 1 | 0 |
| MOL013187 | Chaihu | 0 | 1 | 0 | 0 | 0 | 0 | 0 | 0 | 0 |
| MOL004915 | Gancao | 0 | 0 | 0 | 0 | 0 | 0 | 0 | 1 | 0 |
| MOL004841 | Gancao | 0 | 0 | 0 | 0 | 0 | 0 | 0 | 1 | 0 |
| MOL012269 | Banzhilian | 0 | 0 | 1 | 0 | 0 | 0 | 0 | 0 | 0 |
| MOL005020 | Gancao | 0 | 0 | 0 | 0 | 0 | 0 | 0 | 1 | 0 |
| MOL004907 | Gancao | 0 | 0 | 0 | 0 | 0 | 0 | 0 | 1 | 0 |
| MOL000379 | Huangqi | 0 | 0 | 0 | 0 | 0 | 0 | 0 | 0 | 1 |
| MOL001645 | Chaihu | 0 | 1 | 0 | 0 | 0 | 0 | 0 | 0 | 0 |
| MOL001006 | Dangshen | 0 | 0 | 0 | 0 | 0 | 1 | 0 | 0 | 0 |
| MOL004910 | Gancao | 0 | 0 | 0 | 0 | 0 | 0 | 0 | 1 | 0 |
| MOL008407 | Dangshen | 0 | 0 | 0 | 0 | 0 | 1 | 0 | 0 | 0 |
| MOL000392 | multiDrug | 0 | 0 | 0 | 0 | 0 | 0 | 0 | 1 | 1 |
| MOL004949 | Gancao | 0 | 0 | 0 | 0 | 0 | 0 | 0 | 1 | 0 |
| MOL004996 | Gancao | 0 | 0 | 0 | 0 | 0 | 0 | 0 | 1 | 0 |
| MOL004912 | Gancao | 0 | 0 | 0 | 0 | 0 | 0 | 0 | 1 | 0 |
| MOL000022 | Baizhu | 0 | 0 | 0 | 1 | 0 | 0 | 0 | 0 | 0 |
| MOL004835 | Gancao | 0 | 0 | 0 | 0 | 0 | 0 | 0 | 1 | 0 |
| MOL004974 | Gancao | 0 | 0 | 0 | 0 | 0 | 0 | 0 | 1 | 0 |
| MOL004879 | Gancao | 0 | 0 | 0 | 0 | 0 | 0 | 0 | 1 | 0 |
| MOL001973 | Banzhilian | 0 | 0 | 1 | 0 | 0 | 0 | 0 | 0 | 0 |
| MOL005001 | Gancao | 0 | 0 | 0 | 0 | 0 | 0 | 0 | 1 | 0 |
| MOL001484 | Gancao | 0 | 0 | 0 | 0 | 0 | 0 | 0 | 1 | 0 |
| MOL000497 | Gancao | 0 | 0 | 0 | 0 | 0 | 0 | 0 | 1 | 0 |
| MOL007514 | Dangshen | 0 | 0 | 0 | 0 | 0 | 1 | 0 | 0 | 0 |
| MOL000359 | multiDrug | 1 | 0 | 1 | 0 | 0 | 0 | 0 | 1 | 0 |

TableS2: Genetic characteristics of module1.

| name | Betweenness | Closeness | Degree | Eigenvector | LAC | Network |
| --- | --- | --- | --- | --- | --- | --- |
| STAT3 | 700.4482 | 0.710714 | 238 | 0.145705 | 103.6303 | 205.601 |
| TP53 | 1658.206 | 0.753788 | 270 | 0.1518 | 99.7037 | 240.4924 |
| ERBB2 | 431.9297 | 0.648208 | 186 | 0.118345 | 88.77419 | 138.459 |
| EGFR | 1302.736 | 0.708185 | 236 | 0.138152 | 94.91525 | 194.7362 |
| CASP3 | 659.4057 | 0.70318 | 236 | 0.145489 | 104.3051 | 204.0731 |
| CAV1 | 93.58151 | 0.581871 | 126 | 0.091417 | 79.49206 | 90.65106 |
| BCL2 | 537.5559 | 0.698246 | 228 | 0.142885 | 104.1404 | 193.8756 |
| HIF1A | 983.8105 | 0.698246 | 230 | 0.143123 | 103.7565 | 193.8239 |
| AKT1 | 2005.075 | 0.777344 | 286 | 0.155969 | 98.18182 | 259.3637 |
| MYC | 776.4224 | 0.698246 | 230 | 0.139534 | 99.58261 | 194.8093 |
| CCND1 | 343.8135 | 0.650327 | 194 | 0.126926 | 99.62887 | 157.3436 |
| HSPB1 | 59.93625 | 0.549724 | 88 | 0.066644 | 62.27273 | 65.58074 |
| MAPK3 | 877.0366 | 0.676871 | 212 | 0.130393 | 93.43396 | 165.9461 |
| KDR | 79.67266 | 0.585294 | 124 | 0.091115 | 79.87097 | 89.58552 |
| EGF | 437.1975 | 0.648208 | 190 | 0.124822 | 97.76842 | 153.0356 |
| SOD1 | 177.6618 | 0.555866 | 86 | 0.059499 | 51.34884 | 56.83436 |
| PPARG | 836.3907 | 0.679181 | 212 | 0.129545 | 94.56604 | 168.0275 |
| SLC2A4 | 97.63904 | 0.554318 | 92 | 0.060988 | 52.52174 | 60.10189 |
| GSK3B | 351.3998 | 0.644013 | 180 | 0.116718 | 90.31111 | 135.3537 |
| PGR | 157.1191 | 0.571839 | 114 | 0.076981 | 66.52632 | 77.17879 |
| FN1 | 363.8168 | 0.629747 | 174 | 0.115905 | 93.51724 | 134.4831 |
| PARP1 | 209.1275 | 0.606707 | 150 | 0.103177 | 88.53333 | 112.7821 |
| MCL1 | 59.33862 | 0.580175 | 124 | 0.092245 | 86.19355 | 97.90629 |
| MMP9 | 847.2807 | 0.69338 | 228 | 0.141383 | 102.9474 | 197.3608 |
| BCL2L1 | 141.2547 | 0.616099 | 160 | 0.113747 | 97.55 | 125.6573 |
| ESR1 | 1248.733 | 0.708185 | 236 | 0.140131 | 98.88136 | 197.1765 |
| CCL2 | 351.8517 | 0.637821 | 180 | 0.11617 | 93.06667 | 141.403 |
| PPARD | 74.7761 | 0.542234 | 80 | 0.056098 | 48.3 | 52.48675 |
| PPARA | 508.01 | 0.60303 | 142 | 0.08638 | 67.04225 | 93.96145 |
| MAPK14 | 219.7034 | 0.601208 | 138 | 0.100263 | 86.37681 | 101.3905 |
| IL1B | 1309.146 | 0.721014 | 246 | 0.142055 | 96.39024 | 209.86 |
| NFKBIA | 130.032 | 0.619938 | 158 | 0.115246 | 100.6076 | 123.7937 |
| IFNG | 291.3963 | 0.639871 | 180 | 0.119623 | 96.66667 | 143.6781 |
| IL4 | 138.7873 | 0.60303 | 142 | 0.099192 | 87.26761 | 109.4937 |
| NR3C1 | 129.6165 | 0.573487 | 112 | 0.078728 | 66.21429 | 75.56031 |
| MDM2 | 137.0222 | 0.599398 | 138 | 0.093439 | 81.68116 | 102.7685 |
| ICAM1 | 167.6672 | 0.619938 | 160 | 0.112848 | 97.5 | 126.7059 |
| CDK2 | 212.7802 | 0.576812 | 122 | 0.081757 | 72.2623 | 89.80925 |
| APP | 447.08 | 0.597598 | 132 | 0.090209 | 72.9697 | 88.06112 |
| NOS3 | 104.9577 | 0.576812 | 114 | 0.079084 | 71.36842 | 82.19623 |
| IL10 | 367.2749 | 0.633758 | 174 | 0.114247 | 92.50575 | 136.8424 |
| PTGS2 | 1410.91 | 0.700704 | 228 | 0.137165 | 97.47368 | 186.979 |
| CXCL8 | 312.179 | 0.635783 | 174 | 0.114899 | 93.37931 | 136.5915 |
| AR | 192.022 | 0.578488 | 120 | 0.08109 | 70.2 | 81.62084 |
| ESR2 | 355.9766 | 0.573487 | 108 | 0.074594 | 62 | 70.87242 |
| RELA | 269.1625 | 0.612308 | 160 | 0.111154 | 92.55 | 119.8203 |
| CDK4 | 212.3212 | 0.578488 | 124 | 0.081993 | 70.90323 | 88.46373 |
| HSPA5 | 429.9426 | 0.59403 | 134 | 0.089836 | 72.47761 | 89.27105 |
| NFE2L2 | 497.6629 | 0.608563 | 148 | 0.098114 | 79.45946 | 101.8933 |
| NQO1 | 196.2865 | 0.551247 | 86 | 0.054883 | 46.4186 | 55.48856 |
| HMOX1 | 396.8802 | 0.616099 | 154 | 0.105194 | 88.05195 | 111.8744 |
| MAPK1 | 274.1028 | 0.604863 | 146 | 0.097911 | 79.23288 | 104.5218 |
| MMP2 | 188.5172 | 0.616099 | 162 | 0.114913 | 97.87654 | 126.8105 |
| SERPINE1 | 180.3848 | 0.585294 | 132 | 0.090015 | 79.87879 | 100.1112 |
| MPO | 218.9909 | 0.570201 | 104 | 0.070243 | 62.84615 | 71.52164 |
| CCNB1 | 76.2895 | 0.565341 | 106 | 0.077325 | 77.13208 | 86.78691 |
| IL1A | 180.2516 | 0.616099 | 160 | 0.110169 | 94.15 | 126.9741 |
| VCAM1 | 141.2547 | 0.595808 | 140 | 0.097735 | 87.2 | 108.9728 |
| MMP3 | 109.5192 | 0.560563 | 106 | 0.078428 | 75.01887 | 83.54219 |
| FOS | 639.038 | 0.66113 | 200 | 0.127824 | 96 | 158.4623 |
| CYCS | 938.1623 | 0.641935 | 178 | 0.113759 | 87.01124 | 129.9355 |
| MMP1 | 69.12904 | 0.557423 | 100 | 0.075754 | 74.16 | 80.3837 |
| NOS2 | 117.1987 | 0.557423 | 98 | 0.070991 | 65.22449 | 70.83895 |
| CASP9 | 476.7242 | 0.599398 | 144 | 0.105014 | 94.44444 | 112.4314 |
| CASP8 | 175.3255 | 0.610429 | 156 | 0.111081 | 96.66667 | 123.2952 |
| STAT1 | 192.2852 | 0.616099 | 160 | 0.113104 | 95.8 | 123.5373 |
| PLAU | 57.00003 | 0.554318 | 102 | 0.076341 | 72.86275 | 80.58582 |
| SPP1 | 131.4264 | 0.580175 | 116 | 0.084928 | 76.62069 | 87.47165 |
| CDK1 | 126.4083 | 0.554318 | 106 | 0.074313 | 69.35849 | 79.92167 |
| MAPK8 | 222.0206 | 0.601208 | 140 | 0.100702 | 86.45714 | 104.3166 |
| CDKN1A | 171.506 | 0.60303 | 150 | 0.105158 | 90.72 | 116.2123 |
| IKBKB | 134.1885 | 0.575145 | 116 | 0.08547 | 75.44828 | 84.23601 |
| CCNA2 | 69.3303 | 0.549724 | 96 | 0.068883 | 69.41667 | 78.6777 |
| PRKCA | 85.86211 | 0.554318 | 96 | 0.068997 | 59.33333 | 66.87825 |

TableS3: Genetic characteristics of module2.

| name | Betweenness | Closeness | Degree | Eigenvector | LAC | Network |
| --- | --- | --- | --- | --- | --- | --- |
| STAT1 | 23.604 | 0.848837 | 120 | 0.130087 | 94.46667 | 108.4576 |
| TP53 | 56.96715 | 1 | 146 | 0.149681 | 102.2466 | 144.4852 |
| STAT3 | 53.41604 | 0.986486 | 144 | 0.14838 | 101.8889 | 141.3603 |
| CASP8 | 18.43791 | 0.820225 | 114 | 0.124762 | 92.07018 | 102.3535 |
| MAPK8 | 18.00071 | 0.811111 | 112 | 0.123036 | 90.35714 | 99.12003 |
| EGF | 26.8433 | 0.869048 | 124 | 0.133329 | 96.19355 | 113.7485 |
| ESR1 | 45.56568 | 0.960526 | 140 | 0.14591 | 101.5429 | 135.8239 |
| ICAM1 | 17.444 | 0.829545 | 116 | 0.127584 | 94.62069 | 105.4037 |
| MAPK3 | 45.35014 | 0.924051 | 134 | 0.138781 | 95.64179 | 124.1818 |
| BCL2L1 | 23.57201 | 0.848837 | 120 | 0.129852 | 94.46667 | 108.8456 |
| MMP9 | 54.02469 | 0.986486 | 144 | 0.14822 | 101.6667 | 141.2193 |
| CCND1 | 36.53377 | 0.901235 | 130 | 0.13687 | 96.55385 | 120.5712 |
| HMOX1 | 20.15361 | 0.820225 | 114 | 0.123877 | 91.08772 | 102.2409 |
| RELA | 21.29966 | 0.829545 | 116 | 0.126 | 91.86207 | 103.4715 |
| IFNG | 21.98527 | 0.848837 | 120 | 0.130416 | 95.6 | 109.6547 |
| MYC | 44.9752 | 0.948052 | 138 | 0.143806 | 100.1159 | 132.3772 |
| NFKBIA | 31.61899 | 0.890244 | 128 | 0.136521 | 97.375 | 118.5042 |
| CASP9 | 17.73764 | 0.811111 | 112 | 0.1228 | 90.92857 | 100.3828 |
| AKT1 | 54.10373 | 0.986486 | 144 | 0.148224 | 101.7222 | 141.2917 |
| MMP2 | 30.03731 | 0.869048 | 124 | 0.132255 | 94.51613 | 112.6564 |
| HIF1A | 56.96715 | 1 | 146 | 0.149681 | 102.2466 | 144.4852 |
| FN1 | 27.5295 | 0.848837 | 120 | 0.128588 | 92.26667 | 107.2692 |
| CXCL8 | 16.77366 | 0.820225 | 114 | 0.125322 | 93.26316 | 103.407 |
| BCL2 | 52.46086 | 0.986486 | 144 | 0.14857 | 102.2222 | 141.6492 |
| GSK3B | 32.78412 | 0.858824 | 122 | 0.128566 | 90.68852 | 108.3027 |
| PTGS2 | 50.94723 | 0.973333 | 142 | 0.146738 | 101.1268 | 138.2007 |
| IL10 | 27.79022 | 0.848837 | 120 | 0.127944 | 92.13333 | 108.0419 |
| CASP3 | 56.96715 | 1 | 146 | 0.149681 | 102.2466 | 144.4852 |
| CYCS | 32.01172 | 0.869048 | 124 | 0.131531 | 93.35484 | 111.9384 |
| IL1B | 45.58667 | 0.948052 | 138 | 0.143474 | 99.71014 | 132.2008 |
| EGFR | 34.70505 | 0.9125 | 132 | 0.139942 | 99.39394 | 124.618 |
| MAPK14 | 18.87477 | 0.811111 | 112 | 0.122417 | 89.42857 | 98.56425 |
| ERBB2 | 24.0414 | 0.829545 | 116 | 0.124927 | 89.65517 | 101.7374 |
| PPARG | 38.27499 | 0.9125 | 132 | 0.138812 | 97.63636 | 123.457 |
| CCL2 | 22.32843 | 0.829545 | 116 | 0.125233 | 91.86207 | 104.747 |
| FOS | 39.74977 | 0.924051 | 134 | 0.140689 | 98.56716 | 126.209 |

search strategy:

(“randomized controlled trials as topic” OR “controlled clinical trial*” OR “randomized*” OR “placebo” OR “clinical trial*” OR “controlled trial*“) AND (“decoction” OR “formula” OR “Tang” OR “Traditional Chinese medicine”) AND (“Neoplasms, Hepatic” OR “Neoplasms, Liver” OR “Liver Neoplasm” OR “Neoplasm, Liver” OR “Hepatic Neoplasms” OR “Hepatic Neoplasm” OR “Neoplasm, Hepatic” OR “Cancer of Liver” OR “Hepatocellular Cancer” OR “Cancers, Hepatocellular” OR “Hepatocellular Cancers” OR “Hepatic Cancer” OR “Cancer, Hepatic” OR “Cancers, Hepatic” OR “Hepatic Cancers” OR “Liver Cancer” OR “Cancer, Liver” OR “Cancers, Liver” OR “Liver Cancers” OR “Cancer of the Liver” OR “Cancer, Hepatocellular”).
